# 3D ultra-high resolution seismic imaging of shallow Solfatara crater in Campi Flegrei (Italy): New insights on deep hydrothermal fluid circulation processes

**DOI:** 10.1038/s41598-017-03604-0

**Published:** 2017-06-13

**Authors:** Grazia De Landro, Vincenzo Serlenga, Guido Russo, Ortensia Amoroso, Gaetano Festa, Pier Paolo Bruno, Marceau Gresse, Jean Vandemeulebrouck, Aldo Zollo

**Affiliations:** 1 Department of physics “Ettore Pancini”, University of Naples ”Federico II”, Naples, Italy; 2 Petroleum Institute, Department of Petroleum Geosciences, Abu Dhabi, United Arab Emirates; 3 ISTerre, Université Savoie Mont Blanc, Chambéry, France; 4 Now at Consiglio Nazionale delle Ricerche, Istituto di Metodologie per l’Analisi Ambientale, Tito, Italy

## Abstract

Seismic tomography can be used to image the spatial variation of rock properties within complex geological media such as volcanoes. Solfatara is a volcano located within the Campi Flegrei, a still active caldera, so it is of major importance to characterize its level of activity and potential danger. In this light, a 3D tomographic high-resolution P-wave velocity image of the shallow central part of Solfatara crater is obtained using first arrival times and a *multiscale* approach. The retrieved images, integrated with the resistivity section and temperature and the CO_2_ flux measurements, define the following characteristics: 1. A depth-dependent P-wave velocity layer down to 14 m, with V_p_ < 700 m/s typical of poorly-consolidated tephra and affected by CO_2_ degassing; 2. An intermediate layer, deepening towards the mineralized liquid-saturated area (Fangaia), interpreted as permeable deposits saturated with condensed water; 3. A deep, confined high velocity anomaly associated with a CO_**2**_ reservoir. These features are expression of an area located between the Fangaia, water saturated and replenished from deep aquifers, and the main fumaroles, superficial relief of the deep rising CO_2_ flux. Therefore, the changes in the outgassing rate greatly affect the shallow hydrothermal system, which can be used as a “mirror” of fluid migration processes occurring at depth.

## Introduction

Solfatara is a tuff cone, which was formed between 3.8 and 4.1 ky ago^1^, located 1.5 km NE of the town of Pozzuoli and about 10 km west of the city of Naples, in Southern Italy. The sub-rectilinear NE and SW rims are cut by two normal faults that strike NW-SE, along which deep geothermal fluids can ascend. Outside the crater, two NW-SE striking faults cut the eastern part of the tuff cone^2^.

Solfatara is one of the many volcanoes located within the Campi Flegrei caldera. This is a nested, resurgent caldera, resulting from two large collapses related to the Campanian Ignimbrite (39 ka^1^) and the Neapolitan Yellow Tuff (14 ka^3^) eruptions (Orsi *et al*.^3^). The Campi Flegrei volcanic system is still active, since the last eruption occurred in 1538 A.D. at Monte Nuovo.

Zollo *et al*.^4^ identified a deep, sill-like, mid-crustal magmatic body supplying heat to this volcanic system at a depth of 7–8 km. They have also identified another shallower interface at 2.5 km depth, associated with a discontinuity between the older caldera deposits and a fluid-saturated metamorphic rock layer. Concerning the magmatic source characteristics, Aiuppa *et al*.^5^ proposed two different source models based upon the measures of Campi Flegrei fumarolic gas output. The two main degassing areas are the Solfatara and Pisciarelli, the latter being located 100 m East of the Solfatara. In particular, the authors estimated that the current Campi Flegrei fumarolic sulphur flux is low, whereas the fumarolic CO_2_ flux is surprisingly high for a dormant volcano in the hydrothermal stage of activity. Thus, they proposed that the current CO_2_ output can be supplied by either a large (0.6–4.6 km^3^), deeply stored (>7 km) magmatic source with low CO_2_ contents (0.05–0.1 wt%) or by a small to medium-sized (0.01–0.1 km^3^) but CO_2_-rich (2 wt%) magma, possibly stored at pressures of 100 to 120 MPa.

The caldera has been characterized by periodic episodes of extended, low-rate ground subsidence and uplift, a phenomenon called bradyseism, accompanied by intense seismic and geochemical activity^6^. During the past century, three main episodes of bradyseism occurred in the area: 1950–1952, 1969–1972, and 1982–1984^7^. During the subsidence following the 1984 crisis, a series of small uplift episodes and seismic swarms occurred at the Solfatara. Saccorotti *et al*.^8^ suggest that these earthquakes are likely to be associated with the vibration of a buried cavity filled with a water-vapour mixture at poor gas-volume fractions. The role of fluids in bradyseism has been recognized by many authors [e.g. refs 5, 9–11].

The Solfatara crater is characterized by intense diffuse degassing and fumarolic emissions [e.g. refs 12–14]. Chiodini *et al*.^15^ showed that Solfatara releases about 1500 t/day of volcanic-hydrothermal CO_2_, as a result of diffuse degassing through soil, during which about 3350 t/day of steam condense. This hydrothermal water mixes up with the meteoric one. The energy released by degassing at Solfatara is much higher than the energy released within the caldera during the current period, through other processes such as thermal conduction, earthquakes, and ground deformation. Moreover, the impressive magnitude of diffuse degassing process confirmed the relevance of fluid and heat transport at Solfatara^15^ and prompted further research to improve the understanding of the hydrothermal system feeding the surface phenomenon.

Although the magmatic source is rather deep, the changes in its outgassing rate greatly affect the shallow hydrothermal system processes, which can therefore be used as a constraint for the fluid migration processes occurring at depth^16, 17^.

The effect of deep processes on the shallow hydrothermal system was confirmed by Afanasyev *et al*.^18^ through a simulation of hydrothermal activity at Campi Flegrei caldera. The authors showed that the physical quantities characterizing the deep source, i.e. gas injection rate, gas mixture composition and temperature, strongly control the shallow gas composition, temperature, CO_2_ flux and heat flux monitored at Solfatara.

This justifies the growing interest of the last years in delineating the physical properties of the shallow hydrothermal system of Solfatara, as one of the means to assess the level of potential danger characterizing this crater of the Campi Flegrei volcanic complex.

**Figure 1:**
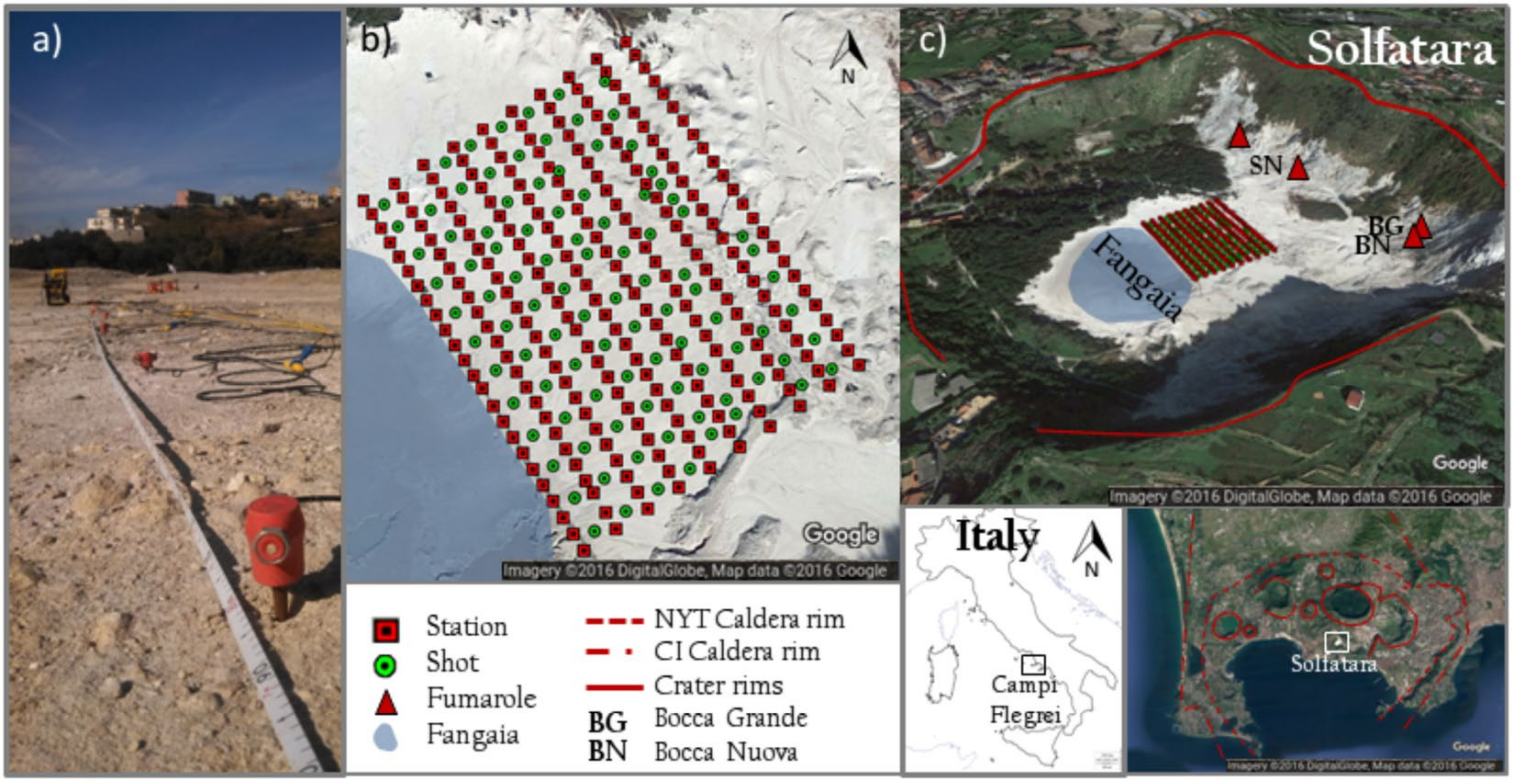
Solfatara with station-shot configuration. (**a**) A photo of the RICEN experiment, with an example of seismic sensors. (**b**) Station-shot configuration. (**c**) The Solfatara crater, with evidence of crater rims, fumarole, Fangaia and the station-shot configuration. The maps in Fig. 1b,c has been obtained with Google Maps 9.38.1 2016 (Map data: Google, DigitalGlobe): *Solfatara, Pozzuoli, Metropolitan City of Naples, Italy* retrieved from https://www.google.com/maps/@40.8174278,14.1393587,1292a,20y,41.32t/data=!3m1!1e3.

In the framework of the experiment RICEN, Repeated Induced Earthquake and Noise (EU Project MEDSUV), an active seismic experiment was carried out between September of 2013 and November of 2014 in order to provide time-varying high-resolution images of the structure of the Solfatara^19^. A grid of 240 receivers, placed at a distance of 5 m on 10 lines, which were 10 m distant from each other, was deployed in the Solfatara crater. A vibroseis energized the soil at the centre of almost all grid cells (Fig. 1). A large and highly informative data-set was then built. The data used in this work were acquired during the first campaign of the experiment, which was carried out on September of 2013.

Velocity and attenuation seismic tomography can be used to image the spatial variation of elastic/anelastic rock properties within complex geological media such as volcanoes. This approach has been widely used in complex tectonic^20–23^, geothermal^24–26^ and volcanic areas^27–31^ in order to detect the presence of fluids and track their migration within the fluid permeated crustal volume.

The goal of the present study is to obtain an ultra-high-resolution (metric) 3D velocity image of the shallow (down to 35 m depth) hydrothermal structure of the central part of Solfatara crater using a technique of delay-time tomography, based on the P-wave first arrival times. Due to the extremely dense acquisition lay-out, the 3D tomographic survey allows us to achieve an unprecedented spatial detail on the shallow velocity structure, which can help in understanding the complex hydrothermal degassing and condensation processes into the porous rocks media. In this way, the interpretation obtained by stratigraphic analysis and resistivity profiles^32, 33^ can be complemented. For our purposes, the results of temperature and CO_2_ flux measurements and of the resistivity survey carried out in the frame of the RICEN experiment will be used to further constrain our interpretation. In fact, several studies over the last few years showed how the multi-parametric analysis of combined geophysical and/or geochemical data sets has been helpful to investigate the complex dynamics of volcanic systems at different scales^34–36^.

In the following, we will briefly describe the data processing and tomographic method, then we will present and analyse the retrieved P-wave velocity model and assess uncertainty and spatial resolution. Finally, we will compare the velocity model with a high-resolution electrical resistivity tomography performed in May of 2014 to investigate and assess the mechanisms of hydrothermal fluid circulation.

### Previous works

Bruno *et al*.^32^ imaged the shallow and intermediate subsurface of Solfatara through the integration of high-resolution geophysical and hydrogeological investigations, including 2D P-wave velocity and Electrical Resistivity Tomography (ERT) profiles. Their results show that the Solfatara subsurface structure can be roughly divided into two zones: a dry, outcropping layer and an underlying saturated zone, in which faults and fractures act as preferential escape conduits for the hydrothermal fluids [refs 1, 32, and 33].

Byrdina *et al*.^37^ used the results from the ERT survey, the mappings of diffuse CO_2_ flux, the ground temperature and self-potential (SP) to understand the mechanisms and paths of shallow fluid circulation. They interpreted the resistivity changes at depth, associated with surface gas flux anomalies, as a double-plume structure: a liquid-dominated conductive plume below the Fangaia mud-pool and a gas-dominated plume below the Bocca Grande fumarole (see Fig. 1).

Isaia *et al*.^33^ integrated the electrical resistivity tomography investigations with the volcano-tectonic information to better constrain the subsurface structure by outlining a complex hydrothermal system. In particular, they assume that the 100 m thick upper zone of Solfatara comprises desegregated rocks and collapse breccias, post-eruptive sediments, whereas the lower sector, down to about 3–4 km, is where the gas-saturated conduit is connected to a magmatic source.

Referring to the Campi Flegrei, de Lorenzo *et al*.^30^ and De Siena *et al*.^31^ carried out two studies on the anelastic properties of the northern part of the Campi Flegrei caldera, including the Solfatara crater. Both studies retrieved a heterogeneous distribution of low-Qp and high-Qp anomalies in the investigated area, thus confirming the strong geological complexity of the subsoil. Moving closer to Solfatara, the two works reveal different features: De Siena *et al*.^31^ found a high Qp and low Qs body. Their correlation with the low Vp/Vs ratio and the low Vp was interpreted as the effect of a small gas reservoir. de Lorenzo *et al*,^30^. on the other hand, found low Qp values at shallow depths, well correlated with high Vp/Vs value^38^; this correlation was interpreted as produced by densely fractured, porous and fluid-filled rocks. A similar interpretation was also provided by Tramelli *et al*.^39^ who found a high-scattering zone in the area of the Solfatara. A recent attenuation tomography^40^ mainly focused on the shallowest subsurface of the Campi Flegrei offshore caldera. The retrieved anelastic images are not spatially correlated with Solfatara; however, they describe the very shallow volcanic system as an environment which is greatly affected by the heterogeneous distribution of different saturation conditions of fluids permeating sediments and rocks of caldera.

Using a subset of the data-bank obtained through the RICEN experiment, Serra *et al*.^19^ found the spatial variation of surface wave phase and group velocities. By inverting the related dispersion curves they obtained a one-dimensional S-wave model for different sub-grids. Together, the different 1-D S-wave models provided a three-dimensional description of the S-wave model in the area down to about 15 m depth. In the upper 4 m, they associated the changes of the S-wave velocity to the temperature gradient, while at greater depths the seismic images were correlated with the resistivity maps obtained from the measurements carried out during the RICEN experiment. They evidenced the presence of a water layer close to the Fangaia area (see Fig. 1) and an abrupt variation in NE direction.

### Data and method

The RICEN experiment consisted in three successive geophysical surveys carried out at the Solfatara volcano respectively in September 2013, May and November 2014, each one lasting one week. During each experiment, the recording both of active seismic data and of the continuous ambient noise was performed. A dataset with more than 75,000 seismograms was collected during the active seismic part of the three experiments. Active seismic data were obtained using a Vibroseis Truck soil energizator, which operated in the frequency range 5–125 Hz. Seismic waveforms were recorded by 4.5 Hz vertical component geophones (GS-11D, Fig. 1a).

In this study, we analyse the data collected during the first experiment. In particular, an area of 90 × 115 m^2^ was sampled by a regular grid of 240 vertical sensors, which were deployed at the crater surface (Fig. 1b,c). The seismic network geometry was set up according to a two-dimensional (2D) grid with 10 lines of 24 sensors, with 5 m spacing between the stations (i.e. in-line distance). The distance between two adjacent lines (i.e. cross-line distance) was 10 m. About 100 shot-points were energized on a staggered grid relatively to the receiver grid. For the vibrational sources, both the in-line and cross-line inter-distances were 10 m. For each shot position, three consecutive energizations were performed and waveforms at each site were stacked in order to increase the signal-to-noise ratio.

**Figure 2:**
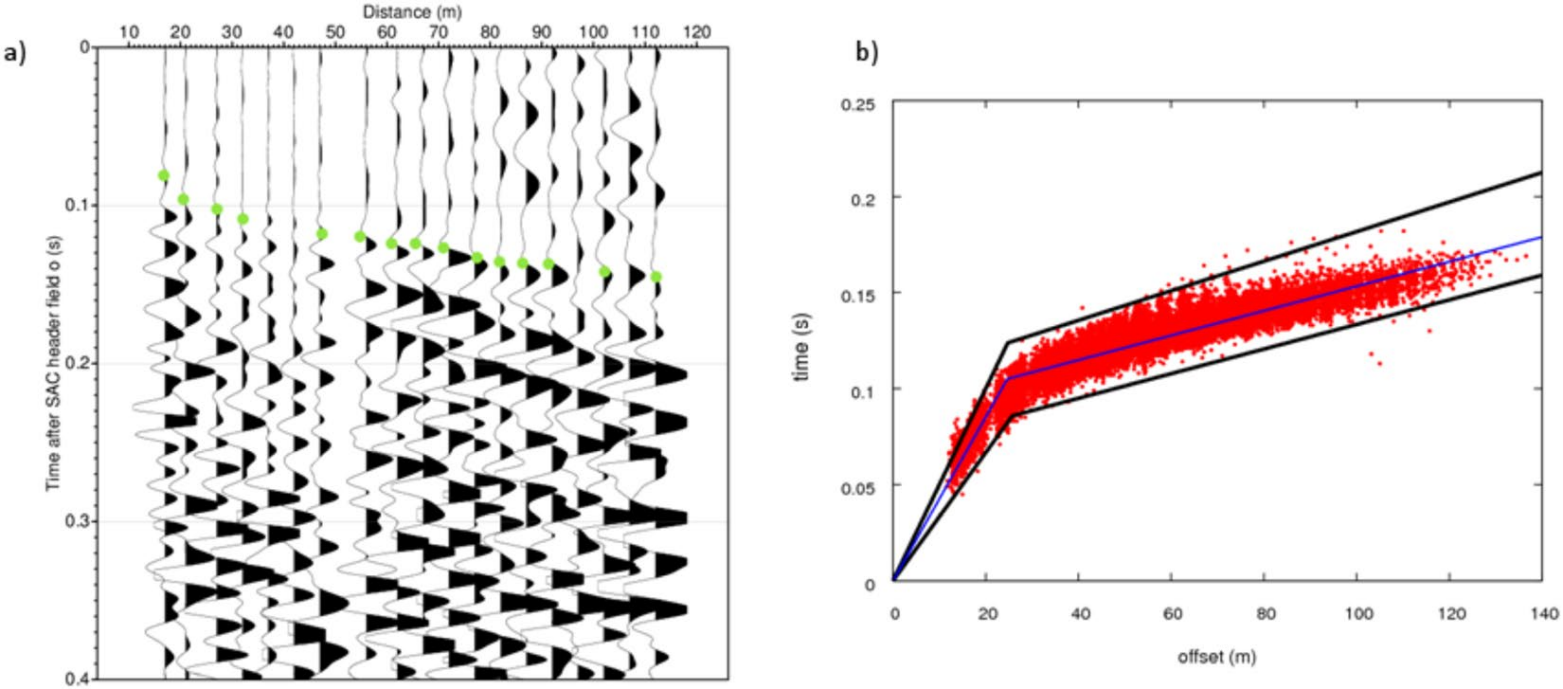
Data and travel-time-vs-distance plot. (**a**) Example of seismic section in common shot gather. The processed traces concerns the shot 194 recorded at stations 25 to 48. The green dots represents picks of first arrival P phases obtained with PROMAX and manually revised. (**b**) Plot of travel-time-vs-distance (red dots). The black lines are the limit imposed for the construction of 1D initial velocity models. Blue line is the 1D initial model selected for the inversion.

It is well known that this volcanic area is very complex, where effects of scattering and attenuation could in general contaminate the first arrival picking. In order to check the quality requirements for an accurate picking analysis, we verified *a posteriori* that the signal-to-noise ratio was sufficiently high at the first arrival onset, and we evaluated waveform/picking coherence vs. offset along the seismic section (Fig. 2a). Through the analysis of the first arrival incidence angles and horizontal polarization directions measured at nearby installed, three-component stations, we confirmed the P-wave nature of the first arriving phase and validated the use of the vertical component for time picking, notwithstanding the strong medium heterogeneity and very short distance between source and receiver In particular, by considering 97 shot records, about the 90% of three-component waveforms showed an incidence angle less than 40° (60% less than 25°) in the first arrival time window, which is the evidence of dominant, near-vertical first arrivals. Moreover, both the horizontal polarization analysis, circular wave-front tracing and apparent velocity estimations, showed a first arrival phase back-azimuth strongly consistent with the expected P-wave azimuth and velocity given the relative location of the shots and the receivers (for further details see Supplementary Materials).

The P-wave first arrivals have been first detected through a Neural Network implemented into the ProMAX SeisSpace software^41–44^ trained on a limited, manually picked dataset of source-receiver couples. In that way about 19,000 picked P-wave travel times have been obtained (for further details check the Supplementary Materials, “Data processing” paragraph). Thereafter, the picking dataset has been manually validated on the basis of a visual inspection of seismic signals. Data affected by a high pick of uncertainty (>0.05 s), based on a very low signal-to-noise ratio (<2), have been discarded. An example of one seismic section in the common-shot gather can be found in Fig. 2a. Moreover, for each shot the travel times referring to the eight closest receivers have been excluded. In those cases, indeed, it was too difficult to correctly measure the P-wave travel time, due to the short pre-event time window available for picking. All data have been considered with the same weight, since only the best quality pickings have been considered for the tomographic analysis. The final selected dataset contained 17,418 P wave travel times relative to 94 sources and 240 receivers.

In order to determine the 3-D P-wave velocity model, the selected travel-time dataset was inverted by applying an iterative, linearized, tomographic approach^45^ and by adopting a *multiscale* procedure^46^. Several inversion runs were performed by progressively increasing the density of grid points (e.g. increasing the model complexity) describing the velocity field, and at each iteration the starting model is the one estimated in the previous run. The proposed procedure is equivalent in principle to moving from a low to a high wave number description of the velocity field. The *multiscale* strategy does not depend on the scale of the application, because its basic assumption is that large wave-length anomalies in the velocity structure have a dominant amplitude when compared to the smaller ones, which we believe is reasonable at all scales of investigation within Earth. This strategy has been already used in seismic tomography^46, 47^ and in migration techniques, where it is known as “multi-scale” approach. The application of this strategy for our analysis is justified by the observed travel-time curves (T(X)) and the distribution of arrival time data vs. offset around their mean trend (Fig. 2b). Indeed, it is clear that even at the small scale of the Solfatara crater, the long wavelength (low-frequency) component of the velocity model (reflected by the piece-wise linear increase in T(X)) has a dominant amplitude relative to short wavelength (high frequency) anomalies possibly producing the dispersion of arrival times around the average trend. We therefore attributed them to both the uncertainty on data picking and to small wavelength perturbations of the velocity model. Specific tests with several inversion, runs with different medium parameterization, confirmed ‘a posteriori’ that the adopted multi-scale strategy was efficient and robust to explore the multi-dimensional model parameter space and to catch the minimum norm model solution (for further details see Supplementary Materials, “Inversion procedure” paragraph).

Due to the source-receiver configuration we have investigated a volume of 160 × 160 × 45 m^3^, the top being at 100 m a.s.l. The three-dimensional hosting medium has been discretized with a grid of regularly spaced nodes.

The final parameterization of the medium in the multiscale procedure is chosen by applying the corrected Akaike Criterion [AICc; ref. 48], which is based on a statistical comparison between models characterized by a different number of model parameters. By introducing the minimum AICc criterion^49^, the problem of selecting the optimal model parameterization is solved avoiding a subjective decision. The minimum AICc value, representing the best compromise between data misfit reduction and model simplicity, is obtained with the 10 × 10 × 5 m^3^ grid spacing (for further details see Supplementary Materials, “Inversion procedure” paragraph), which is the final parameterization in the *multiscale* procedure.

At first, for the *multiscale* approach, a coarser parameterization was used with a node-spacing of 16×16 × 7.5 m^3^.

In order to take into account the possible dependence of the final tomographic solution on the starting velocity model we adopted the following strategy: (1) first, we estimated a reference 1D velocity model; (2) starting from this, we generated a set of 200 1D initial models; (3) a 3D inversion is performed for each initial model.

The estimation of the 1D reference velocity model is obtained by a modelling procedure, which minimized the RMS of the travel time residuals (see Supplementary Materials). This procedure allows us to assess that the simplest velocity model minimizing the travel-time residuals is two-layered structure. As for the 200 1D initial models, they have been constructed as 2-layer models with the depth of the interface fixed to that of the reference 1D velocity model (10 m). The variability range of the velocities in the 2 layers was established by constraining the respective theoretical travel-time curve to lie within the limits defined by the scattering of measured data in a travel-time-vs.-distance graph (see Fig. 2b and Supplementary Fig. S8b). Each 1D velocity model is used as a starting model for the data inversion. Then, by computing the average of the 200 final three-dimensional velocity models and the normalized standard deviation for each model parameter (σ/*V*_*P*_), we observe that deviations from the average model on retrieved velocity values are less than 15%, except for some grid nodes (see Supplementary Fig. S9).

The starting velocity model minimizing the final misfit of the residuals is chosen as the initial velocity model for the inversion with the coarser parameterization (16 × 16 × 7.5 m^3^) (see Fig. 2b). The three-dimensional P-wave velocity model retrieved at the sixth iteration is used as starting model for further data inversion in the final grid (spacing 10 × 10 × 5 m^3^).

For each parameterization we selected a damping parameter using an empirical approach, by performing several inversions with different values^50^. The selected damping parameters are the ones providing the best compromise between the variance reduction of the residuals and the increase of the solution variance. By inspecting the trade-off curves retrieved for each parameterization, the value of 0.5 was selected for all the parameterizations graph (see Supplementary Fig. S10b-c).

In order to assess the reliability of the final solution, we numerically computed the resolution matrix from which we extracted the RDE (Resolution of Diagonal Elements) and the spread function^51^. The results of the resolution analysis allowed us to assess that the resolved area is about 100 × 120 × 35 m^3^ (for further details see Supplementary Figs S13 and S14).

## Results

**Figure 3:**
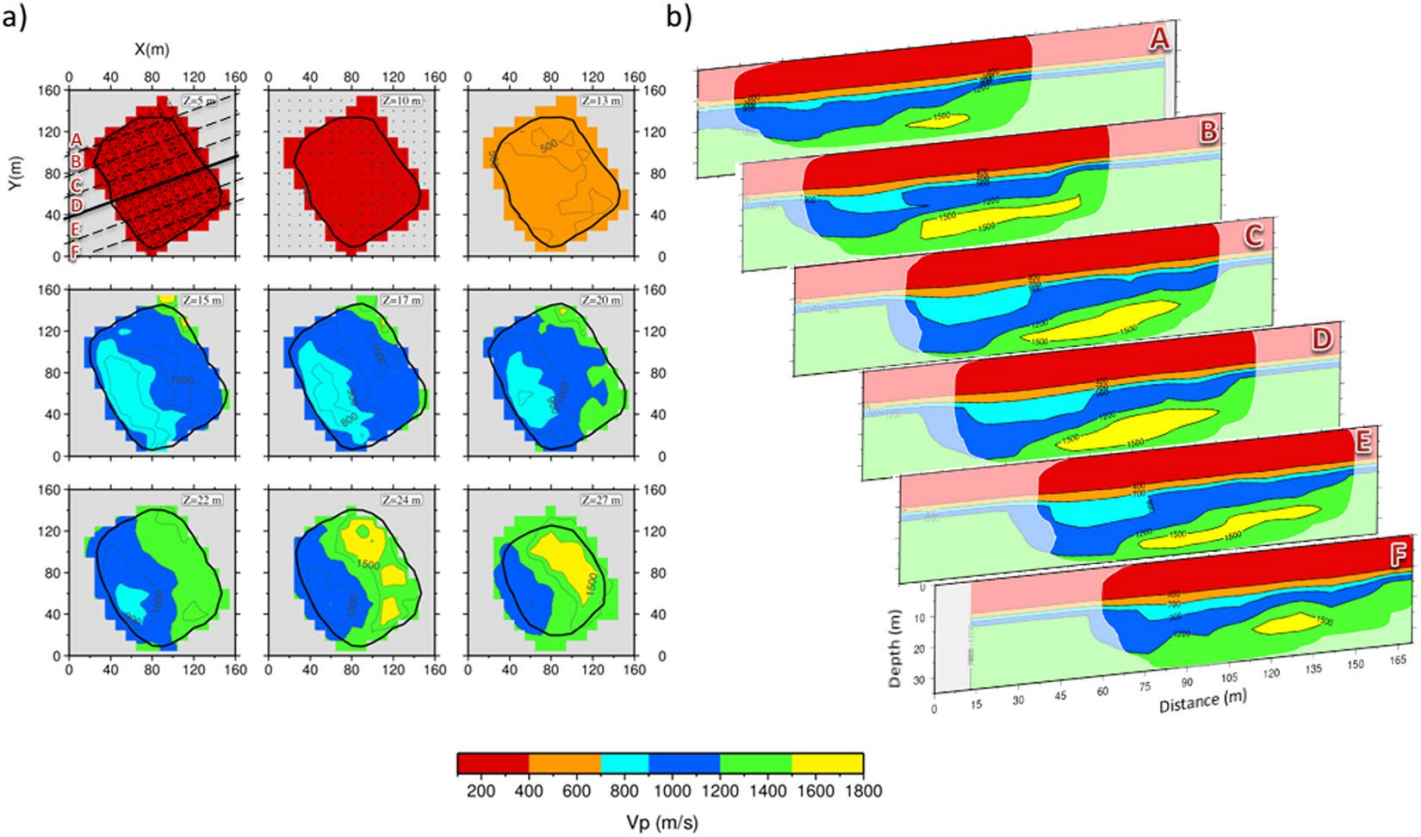
P-wave velocity model. (**a**) Horizontal slice of P-wave velocity model at different depths. The black contour delimitates the resolved area, i.e. the area for which the tree resolution parameters (RDE, Sj and *DWS*) are included in a threshold value. The threshold values of *S*_*j*_ and DWS are chosen in order to obtain a similar contour, binding the RDE to be higher than 0.9. The grey regions in each slice represent areas not covered by rays. (**b**) P-wave velocity model projected onto the SW-NE cross-sections located in Fig. 3a. The blurred regions in each slice represent areas not resolved.

In Fig. 3 we show the 3D P-wave velocity model obtained with the finest parameterization (10 × 10 × 5 m^3^). Considering all the *multiscale* steps, we achieved a reduction of the root mean square of travel times residuals (rms) of about 70%, with a final rms of 4 ms (see Supplementary Fig. S11).

The resolution matrix and the spread function, shown in Supplementary Fig. S12, allow us to state that the final model is well resolved down to 30–35 m depth.

The plane view of the model (Fig. 3a) shows a depth-increasing P-wave velocity structure within the shallower 13–14 m depth, with velocity values ranging from 200 to 700 m/s. Between 13 and 15 m depth an abrupt increase of velocity is observed, from 500 m/s to 1000 m/s. Moreover, around these depths the model shows a significant lateral variation. From 15 m to 22 m depth, in the western part of the model, a low velocity anomaly trending NW-SE is imaged. Its P-wave velocity is about 800 m/s and its areal extension decreases with depth. From 22 m to 30 m depth, a NW-SE interface separates two distinct zones characterized by different compressional velocity values. The Western depth section has a velocity ranging between about 900 m/s and 1200 m/s, and the Eastern one has higher velocity values of about 1200–1500 m/s. Finally, a well confined, lens-shaped, high P-wave velocity zone is imaged in the deepest part of the model, with velocity values higher than 1500 m/s.

The SW-NE cross-sections shown in Fig. 3b allow us to better delineate the shape and areal extension of the features which were already identified in the plane view representation. First of all, the intermediate layer, with velocity ranging between 1000 and 1100 m/s, progressively deepens toward SW. Moreover, it quickly rises again at the interface with the mud pools (Fig. 3b). Then, the low velocity anomaly, included in the previous layer, is more extended in the central part of the model (see section B-C-D in Fig. 3b), and reduces towards the northern and southern edges of the grid (see section A-E-F in Fig. 3b). Finally, the same behaviour is observed in the deep high velocity anomaly, which becomes thinner and thinner at the edges of the grid and completely disappears in the “F” section in Fig. 3b.

## Discussion

In this study we obtained a new 3D, high-resolution image of P-wave velocity of the Solfatara crater through an inversion strategy based on a *multiscale* approach. We suggest that the retrieved subsoil velocity images can be explained in terms of the interaction between structural patterns and degassing dynamics within shallow hydrothermal circulation cells^32^. According to the geochemical analysis of the Solfatara fumarolic gases performed by Caliro *et al*.^52^ this degassed flux is composed by deep magmatic CO_2_-rich fluids mixed with hydrothermal liquids of meteoric origin.

The Solfatara shallow stratigraphy consists of eruption deposits, *tephra*, which are hydrothermally altered and mainly composed of alternating fine to coarse ash deposits with limited distribution, scoria layers, and lavas^33^. In detail, the first 10–15 m are composed by recent, unconsolidated deposits; beneath this layer, the deeper 20–30 m thick deposits have a dominant composition of Astroni tephra, i.e. sandwave, ash surge and fallout deposits with variable degrees of consolidation^33^. The retrieved velocity values, V_p_ < 1800 m/s, are consistent with the ones found in other volcanic areas for the tephra deposits^53, 54^. In particular, in the first 10–15 m the P-wave velocity values range from 200 m/s to 700 m/s, which correspond to aerated tephra^54^. On the other hand, the higher velocity values in the deeper zone (V_p_ up to 1800 m/s) can be related to the tephra deposit, which are more consolidated and possibly saturated^53, 54^.

**Figure 4:**
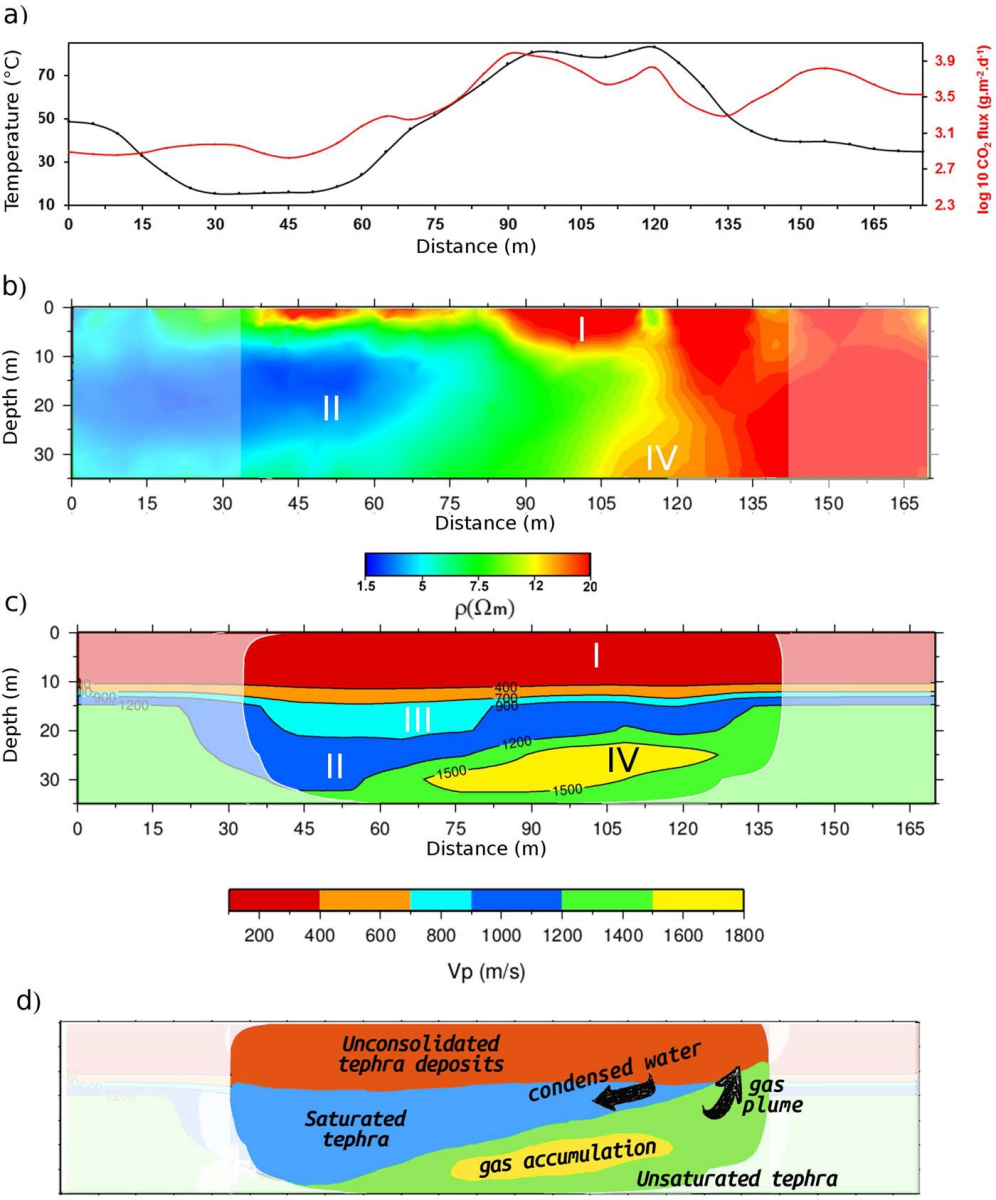
Comparison between temperature (black line) and CO_2_ flux (red line) in (**a**), resistivity cross section in (**b**) and P-wave velocity model projected onto the cross-section D in Fig. 3a in (**c**). (**d**) Schematic representation of geological features and fluid flux direction, discussed in the interpretation.

Taking into account that the presence of fluids and their circulation may greatly affect the rock volume, and therefore the average compressional wave velocity, we expect that the tomographic images can constrain the possible location and phase of permeating fluids^55^. For this purpose, we compared our seismic tomography with 2D cross sections of resistivity (ρ) and with temperature and CO_2_ flux measurements. Serra *et al*.^19^ show how the integration of velocity images with resistivity tomography provides a more complete interpretation of the complex Solfatara system. A high-resolution electrical resistivity tomography was performed with 16, 115-m-long NW–SE profiles and 24, 75-m-long NE–SW profiles. The surveys were carried out in March and May 2014 during the RICEN experiment. We used a Wenner-Schlumberger configuration with a 5 m spacing between electrodes. In addition to these short profiles, we used the 1 km-long profile performed by Byrdina *et al*.^37^ (labeled Pr2 in their paper) in order to increase the resolution at depth. Resistivity data were filtered by removing values with a standard deviation exceeding 5%, or when the injected current was lower than 20 mA. The 3-D resistivity inversion was performed with 3028 filtered apparent resistivity measurements using RES3DINV software^56^ which adopts a smoothness-constrained least-square algorithm^57^. The resistivity tomogram shown in Fig. 4a has a 7.5% rms error after 5 iterations. The same section of the sensitivity map, which shows a very good resolution up to 25–30 m depth, has been provided in Supplementary Fig. S15. The electrical conductivity can be written as the sum of the surface conductivity, which is prominent in the case of clay-rich minerals produced by hydrothermal alteration, and bulk conductivity, which in our case mainly depends on hydrothermal fluid saturation and temperature^58^. The CO_2_ flux has been measured using the Accumulation Chamber Method [for details, see ref. 59], whereas the soil temperature was recorded at 30 cm depth by using a type K thermocouple. Both measurements have been carried out at each electrode location during the electrical resistivity tomography survey. The studied area includes the second largest diffuse degassing spot at Solfatara after the fumarolic area, with CO_2_ flux values ranging from 700 to 10000 g.m^−2^.day^−1^. This high CO_2_ flux anomaly is accompanied by a significant thermal anomaly, with soil temperature up to 80 °C at 30 cm depth, due to the latent heat transfer that occurs during steam condensation at the surface.

The resistivity values of the SW-NE cross-section range between 1.5 and 20 Ωm, thus indicating a globally conductive subsurface for Solfatara (Fig. 4b), in agreement with the previous results^33, 37^. The model clearly highlights a sharp horizontal contrast between a resistive structure (20 Ωm) beneath the eastern part of the cross section and a conductive body (<5 Ωm) on the western part, towards the liquid-saturated Fangaia mud pool.

With reference to Fig. 4c, the superficial layer “I”, with a P-wave velocity lower than 700 m/s, can be related to the shallow resistive zone “I” in Fig. 4b, with resistivity ρ > 12 Ωm, located at the top of the resistivity section. The same resistive upper layer was obtained by Byrdina *et al*.^37^ and can be interpreted as unsaturated recent deposits^32, 33^. This layer is affected by a high CO_2_ degassing (Fig. 4a), higher than 1000 g.m^−2^d^−1^, and by an elevated surface temperature (>40 °C, Fig. 4a). A passive seismic survey performed by Letort *et al*.^60^ identified this area as the source of the ambient seismic noise generated by hydrothermal processes. The 3D velocity sections (Fig. 3b) allow us to delineate this layer, which extends through the entire central area of the crater.

The second layer “II” shows P-velocity values ranging from 900 m/s to 1200 m/s and is about 10 m thick. It progressively deepens toward the SW part of the crater, becoming shallower at the interface with the mud pool (Fig. 4c). This structure corresponds to the eastern part of Fangaia, an area saturated with mineralized liquids^37^. Here, the resistivity image shows at 10–30 m depth a body (“II”) saturated with conductive liquid (Fig. 4b),with ρ  < 5 Ω m, getting deeper in the Fangaia direction like the velocity layer “II”. The slope of the liquid-saturated body is explained by a pronounced influence of local topography^37^.

On these grounds, we suggest that steam condensation is produced on the eastern part (“IV” and above) in a gas dominated structure that is characterized by both high diffuse degassing and high surface temperature. This steam produces hot condensate water, which is channelled within the “II” layer and finally reaches the Fangaia mud pool in the western part.

This fluid directionality is also inferred from the Self-Potential mapping by Byrdina *et al*.^37^ Indeed, in their paper Self Potential anomalies decrease from −40 mV in the eastern part to −100 mV in the Fangaia area. In a volcanic area, the streaming potential is the main source of current. It is associated with the drag of the excess of charge in the pore water of fluids. Consequently, above the isoelectric point of pH (which is the case in the Fangaia survey, with a soil pH above 5), an advective flow can be inferred from a decrease of the streaming potential.

In Fig. 4c the low velocity anomaly “III”, with values between 700 and 900 m/s, is characterized by the same conductive properties already described for the body “II” in Fig. 4a. There, we expect to find a region saturated with the liquid flow of the Fangaia area. However, inasmuch this body has velocity values lower than the “II” layer, we suggest, in agreement with the lithology^33^, that it is composed by less consolidated tephra sediments than the layer “II”. Therefore the velocity variation between the “III” and the “II” zone may identify a gentle discontinuity in the degree of consolidation of rocks.

The shape of this anomaly, which has the maximum extension in the central area and decreases going towards the NW and SE directions (Fig. 3b), could be due to the accumulation of deposit materials in a low of the most compact layer “II”.

The previous results are in agreement with what was found in Serra *et al*.^19^ The retrieved 3D S-wave velocity model, obtained from a combination of 1D velocity models in array sub-grids and resolved up to 15 m, showed two main domains: the S-W one, closest to the Fangaia, is slower than the N-E one. This feature is common to all the depths. In order to interpret the low S-wave velocity domain at SW, the authors qualitatively hypothesized a manifestation of an unconsolidated layer at shallow depths and a water aquifer at greater depths. The P-wave velocity model obtained in this work not only has a much higher resolution, but also covers an area that is about twice the one of the S-wave velocity model obtained by Serra *et al*.^19^ These features allowed us to be in agreement with the previously results, but also to considerably increase the knowledge and characterization of the deeper anomalies of the Solfatara hydrothermal system. The water aquifer, mentioned by Serra *et al*.^19^ is imaged and better characterized through the interpretation of layer “II”, located at 15–20 m depth.

Finally, we will try to interpret the high velocity anomaly “IV” in Fig. 4c. It is located between 25 and 30 m depths and it constitutes a novelty with respect to the S-wave velocity model by Serra *et al*.^19^ since no resolution was achieved in this cited work. This high velocity anomaly is characterized by values that range between 1500 and 1800 m/s. The anomaly “IV” does not have a perfect match in the ERT section, but could be located in resistivity tomography at depth between the resistive and conductive bodies. The high-velocity body appears at the beginning of the degassing structure (Stufe di Nerone, SN in Fig. 1) on the eastern border of Solfatara crater, where major NW-SE directed fractures^33^ enhance the up-flow of the water steam and the CO_2_ flux. This interpretation is consistent with the high CO_2_ flux and ground temperature measured in the same area^37^.

From the resistivity images (Fig. 4b) it can be clearly seen that the conduit of the rising gas plume on the NE direction is located where the “II” layer saturated with condensed water is thinner (Fig. 4c). Therefore, the anomaly found in our velocity images can represent an area of gas accumulation, trapped by the liquid saturated “II” layer, located immediately above and not completely resolved by the resistivity images due to its location below a conductive area.

In order to summarise our interpretation, we provide in Fig. 4d a schematic representation of the discussed features and the direction of gas and condensed water fluxes.

The velocity tomographic model obtained for the Solfatara allows us to image with a high resolution the shallow area of the crater and to understand the processes taking place inside. These processes are part of a complex dynamics triggered by the interaction between structural patterns and degassing within shallow hydrothermal circulation cells. Then, at the shallow investigated depths, we can see the effect of deep processes affecting the caldera, and our investigated area can be interpreted as the surface evidence of deeper processes.

## Conclusion

The importance of this work lies in these principal aspects:

1. the Solfatara crater represents one of the main pressure release areas of the entire Campi Flegrei volcanic system, considering the impressive magnitude of the diffuse degassing process. Hence, the interest in the knowledge of this area grows, especially with the aim of assessing the level of potential danger characterizing this crater;
2. the 3D tomographic survey allows us to achieve an unprecedented spatial detail on the shallow velocity structure of the central part of the Solfatara crater. The 3D high-resolution tomographic images allow us to better understand, in terms of velocity anomalies and fluid type, the complex hydrothermal processes into the shallow part (30–35 m) of the volcano;
3. the procedure used in this work represents a new multi-parametric approach that can be used in a volcanic environment; it shows how the interpretation of velocity tomographic images can be complemented with the ones obtained by stratigraphic analysis and resistivity profiles, and, most of all, how this joint interpretation leads to a more robust and reliable interpretation of complex hydrothermal system;
4. the complex interactions (deep fluids, hydrothermal system, geological structures) between deep and shallow sources and structures allow us to use the shallow hydrothermal system processes as a constrain for the fluid migration processes occurring at depth.

## Electronic supplementary material

Supplementary Material
