# Supplementary material for: 3D ultra-high resolution seismic imaging of shallow Solfatara crater in Campi Flegrei (Italy): New insights on deep hydrothermal fluid circulation processes
Source: Sci Rep. 2017 Jun 13;7:3412. doi: 10.1038/s41598-017-03604-0 (PMC5469761; doi:10.1038/s41598-017-03604-0)
Supplement: Supplementary file 1 — Supplementary Material [file 41598_2017_3604_MOESM1_ESM.pdf]

Supplementary Material of

3D ultra-high resolution seismic imaging of shallow Solfatara crater in Campi Flegrei(Italy): New insights on deep hydrothermal fluid circulation processes

Grazia De Landro*[1], Vincenzo Serlenga[14], Guido Russo[1], Ortensia Amoroso[1], Gaetano Festa[1], Pier Paolo Bruno[2], Marceau Gresse[3], Jean Vandemeulebrouck[3] and Aldo Zollo[1]

(1) University of Naples “Federico II”, Department of physics, Italy.
(2) Petroleum Institute, Department of Petroleum Geosciences, Abu Dhabi, United Arab Emirates
(3) ISTerre, Université Savoie Mont Blanc, Chambéry, France.
(4) Now at Consiglio Nazionale delle Ricerche, Istituto di Metodologie per l’Analisi Ambientale, Tito, Italy
Corresponding author: Grazia De Landro (delandro@fisica.unina.it)

Data processing

We used a Neural Network implemented into ProMAX SeisSpace software [1,2,3,4] to pick preliminary first arrivals on the entire 3D dataset. We first created a training dataset by manually picking the first breaks and saving them into a database file. Since our dataset has not a large variance in character from one end to the other, we only used two shots picked and checked for consistency on all inline and crossline profiles. The number of training shots was kept intentionally low since the more the shots used for training, the longer the learning time, and the ability of the neural net to learn can be degraded [3].
The neural net uses this training dataset to learn the correct first break picks on the traces in the picked gathers. When the learning phase is completed, a table is created to store the network’s topology and its weight matrix. This weight file was then used to automatically pick the first breaks for the rest of the dataset.
A key to success with the neural net training and picking is the guide function in the form of upper and lower gate limits. These will be used to limit the segment of the data in which the program will search for first breaks. A good rule which we followed is to allow two periods above the first break to start the gate and about that same amount below the first break for the bottom of the gate. The guide function was picked on each shot to provide consistent placement of the first breaks for all shots.
First arrival picks provided only a preliminary basis for a subsequent manual revision of the entire dataset. The revision was performed in two stages and by two different groups in order to minimize subjective picking. In correcting the picks, we projected them over different data gathers (i.e. Common Shot, Common Receiver and Common Offset) and we strictly followed the reciprocity rules described by Ackerman [5]. Large redundancy within the data allowed us to greatly reduce the ambiguities in the picked traveltimes. Uncertainty on travel time readings ranges from a minimum of 0.005 ms to 0.05 ms on large offsets on noisy data.
Data in input to the Neural Network picking was not stacked (i.e. we used a single-source single geophone acquisition scheme) and had a just a minimal processing applied which is described below:

I. Vibroseis correlation using the Filtered Ground Force (FGF) pilot sweep. This FGF signal is generated in the SIB-100 box in MiniVIB truck real time by combining the baseplate accelerometer and the mass accelerometer signals generated during each sweep. It is a true indication of the vibrator phase and amplitude performance during each sweep. This signal is then filtered with a tracking high cut filter. The frequency of this tracking filter is set to remove all higher order harmonics.
II. Reduction of effect of minimum-phase ground filtering: vibroseis crosscorrelation produces a Klauder (i.e. zero-phase) wavelet that is then convolved with the minimum-phase earth filtering [6,7,8] producing a mixed-phase wavelet. In spiking deconvolution there is an assumption that the embedded wavelet is minimum-phase. For vibroseis data this incorrect assumption creates a phase rotation in the data. To remove the mixed phase wavelet we followed the approach of Gibson and Larner's [7]. We estimated the minimum-phase equivalent of the Klauder wavelet by using the FGF pilot sweep. The associated inverse filter is used to convert the embedded zero-phase wavelet in the trace after cross-correlation to minimum phase. This allows to improve the picking of first arrivals and for reflection data also allows an accurate application of minimum phase spiking deconvolution.

Promax toolbox, through the previously described procedure, provided only preliminary picks which were then manually validated.
Concerning the visual inspection, data characterized by a picking uncertainty greater than 0.03 s have been discarded. After this preliminary selection, we conclude that, on average, we removed traces characterized by a picking uncertainty of 0.05 s.
The comparison of spectra of the first P-wave in a time window of 0.128 sec at different offsets with the pre-event noise spectra (see figure S1) shows that up to frequencies of about 80 Hz, the signal-to-noise ratio is relatively high, thus it guarantees a good quality recording of the first P arrival at 4.5 Hz velocity sensors.

Also the S/N ratio has been adopted as criterion to discard travel time data from dataset. In particular, we compared the maximum amplitude before and after the identified P-wave arrival time. We performed this analysis in two equivalent 0.2 s wide time windows before and after the pick. All data characterized by a S/N ratio approximately less than 2 have been discarded.

Finally, we removed from dataset provided by automatic picking by ProMAX, all data for which a trivial picking by eye is completely impossible. In that way, we performed a very restrictive selection which allowed us to invert only travel time data referring to traces for which the P-wave arrival time is very clear on the seismic signal.

Picking reliability

To ensure that in such attenuating medium and even at short distances, most of the direct energy produced by the source is not lost lost via scattering and absorption, we performed a spectral analysis. Although in the frequency band of the source (5-125 Hz) P-wave energy can be lost in anelastic attenuation, surface wave conversion and scattered field, the comparison of spectra of the first P-wave in a time window of 0.128 sec at different offsets with the pre-event noise spectra (see fig. S1) shows that up to frequencies of about 80 Hz, the signal-to-noise ratio is relatively high (~10). This guarantees a good quality recording of the first P arrival. Finally, it is worth to note that the selection of the sampling rate of 1KHz ensures an anti-aliasing filter cut-off related to the data-logger well beyond the maximum frequency radiated by the Vibroseis source.

Moreover, in order to validate the use of the vertical components in our case, we performed an analysis of particle motion direction. We used a three-component station (green square in fig. S2) located near one of the array geophones (blue rhombus in fig. S2, distance is about 5 m). We considered the waveforms of the 97 shots recorded at this station (fig. S2, blue diamond). Using the method of Rosenberg et al [9], based on SVD decomposition, we calculated the cosine of the angle (from vertical) of the direction of particle motion (s_n). Then, we considered the maximum of s_n in a 0.2 seconds time window centered on P pick (fig. S3). The histogram of these values (fig. S4) shows that about the 90% of waveforms has an incidence angle of particle motion less than 40° around the P-wave arrival time, in particular, about the 60% has an incidence angle less than 25°. Indeed, we are able to identify the P-wave first arrival on vertical components.

In addition to the value of incidence angle of the wave around first arrival, another strong evidence that the identified phase corresponds to a P wave is that the retrieved range of velocities in the tomographic image is comparable with that one typical of for tephra materials [10, 11]. These latter are the main component of the shallow structure at Solfatara [12].

Furthermore, to corroborate that the recorded phase corresponds to a P wave, we performed a polarization analysis that allows to compute the polarization attributes from the eigenvalues and eigenvectors of the covariance matrix [13] implemented in the SeisGram2k code (http://alomax.free.fr/seisgram/SeisGram2K.html). For the analysis, here too, we considered the traces of shots recorded at 3-component stations represented with a red triangle in fig. S5 and calculate the polarization attributes in the time window around the P first arrival (fig. S6, left panels). Some example of the obtained results are showed in fig. S6. The results clearly show that the recorded waves are polarized along the propagation direction (azimuth corresponding at station-shot direction), typical feature of P-waves.

Finally, another evidence about the nature of the recorded first arriving waves derives from tracing the first wave-front propagating across the array for a given shot and the determination of shot position (distance between shot and a reference receiver), azimuth (as measured for a central array reference receiver) and apparent velocity through the inversion of the observed differential arrival times. The array location technique is based on circular-wave-front (CWF) approximation so, it is very useful in volcano seismology analyses given the short distance between source and receivers [more details can be found in 13 and 14]. The result of this analysis applied to several shots provided very consistent shot locations and azimuths (within 2 meters and few degrees from true location and azimuth), validating the coherence of P phase picking on the entire array configuration, and an apparent velocity of 900-1000 m/sec. In particular this apparent velocity value matches well the expected P-wave velocity propagation in the shallow crater subsurface, and it is much smaller than S and surface wave velocities as retrieved from the modelling and imaging of surface waves [16]. In fig. S7 we show the results of the application at shot 132.

Dependence analysis on the choice of initial model

In fig. S8 we show the 200 1D initial models constructed as 2-layer models with the depth of the interface fixed to that of the reference 1D velocity model (10 m depth). Each 1D velocity model is used as a starting model for the data inversion in the coarser parameterization 16x16x7.5 m^3. In fig. S8a we show the distribution of final (in green) and initial (in red) rms of the inversion with the 200 2-layer velocity models. This distribution shows a reduction of about 70% of rms associated to initial and final model.

Then, we computed the average of the 200 final three-dimensional velocity models and the normalized standard deviation for each model parameter (σ/V_P). In fig. S9 we show the standard deviation of average model, observing that deviations from average model on retrieved velocity values are less than 15%, except for some grid nodes.

Inversion procedure

The tomographic inversion is performed applying an iterative, linearized, procedure described in Latorre et al. [17], but the reviewer is right, the multi-scale approach that we use here is similar to the one adopted by Zollo et al. [18]. However, this strategy does not depend on the scale of the application, provided that data have controlled uncertainty and sufficient resolution to retrieve unbiased model parameters. The basic assumption is that large wavelength anomalies in the velocity structure have a dominant amplitude relative to the smaller ones, which we believe is reasonable at all scales of investigation within Earth. The initial inversion runs are performed using a low-resolution, high-wavelength parameterization of the medium, which is progressively refined by increasing the density of grid points at successive runs. We believe that this strategy is justified by the observed travel-time curves (T(X)) and the distribution of arrival time data vs. offset around their mean trend (fig. 2b of main text). Indeed, it is clear that even at the small scale of the Solfatara crater, the long wavelength (low-frequency) component of the velocity model (reflected by the piece-wise linear increase in T(X)) has a dominant amplitude relative to short wavelength (high frequency) anomalies possibly producing the dispersion of arrival times around the average trend. We therefore attributed them to both uncertainty on data picking and small wavelength perturbations of the velocity model.
Specific tests with several inversion runs with different medium parameterization, confirmed 'a posteriori' that the adopted multi-scale strategy was the more efficient to explore the multi-dimensional model parameter space and to catch the minimum norm model solution. The comparisons of histograms of travel time residuals computed for the initial (coarse) and the final (fine) velocity models is another evidence for it (fig. S11). The finest parameterized velocity model produces a more peaked histogram of residuals centred at zero (e.g. a smaller data dispersion around the a zero-mean value) than the coarse parameterized model.

The final parameterization of the medium in the multiscale procedure is chosen by applying the corrected Akaike Criterion [$AICc$; 19, 20], which is based on a statistical comparison between models characterized by a different number of model parameters. For each parameterization (16 x 16 x 7.5 m^3, 10x10x5 m^3, 10x5x5 m^3), the $AICc$ value is computed in the following way:

$$AICc = 2k + nlog\left(\frac{rms}{n}\right) + \frac{2k(k+1)}{n-k-1}$$

In the previous equation k represents the number of model parameters, whereas n represents the number of data.
The minimum $AICc$ value, representing the best compromise between data misfit reduction and model simplicity, is obtained with the 10x10x5 m^3 grid spacing, which is the final parameterization in the multiscale procedure. By introducing the minimum $AICc$ c criterion, the problem of selecting the optimal model parameterization is solved avoiding a subjective decision.
In fig. S8a is shown the rms reduction vs the iteration using the *multiscale* approach with the two parameterizations. Furthermore, in fig. S9 are shown the histograms of inversion residuals for the finer parameterization 10x10x5 m^3. The final residuals histogram shows a tight distribution centered at zero.

For the calibration of the damping factor we followed an empirical approach: using real dataset, we performed several inversions for different values of damping. The data variance and solution variance are computed after 6 iterations for indicated damping values. The chosen damping value is the one in which a small variance in the data corresponds to a small variance of the model.
The L-curve representing the data variance in function of model variance are plotted in fig. S10b, for the 16x16x6.5 m^3 parameterization and in, fig. S10c, for the 10x10x5 m^3 parameterization. In both cases, the chosen damping value is 0.5.

Velocity resolution analysis

The full resolution matrix is calculated starting from the tomographic matrix using the relation 11 of Rawlinson and Spakman [21]. The full resolution matrix is represented in terms of its resolution diagonal elements RDE and the spread function S_j [22] related to off-diagonal elements. In particular, the S_j is defined as:

$$S_j = log\left(|s_j|^{-1}\sum_{k=1}^{N}\left(\frac{s_{kj}}{s_j}\right)^2 D_{jk}\right)$$

where s_j is the L_2 norm of diagonal j element of resolution matrix, and can be interpreted as a weighting factor that takes into account the value of the resolution kernel for each parameter, s_{kj} is the elements of j-th row of resolution matrix, and D_{jk} is the distance between model parameter j and k. So, S_j is calculated by compressing each row of the resolution matrix into a single number, that describes how peaked is the resolution for the corresponding diagonal element. The lower the S_j the more peaked is the resolution.

We used for the definition of resolved area, in addition to RDE and S_jj, the derivative weight sum (DWS) that measures the ray density in the neighborhood of every node of the tomographic grid [23, 24]. The DWS of the nth V parameters is defined as

$$DWS(V_n) = N\sum_i\sum_j\left\{\int_{L_{ij}} \omega_n(x)ds\right\}$$

where i and j are indices for event and station, ω is the linear interpolation weight that depends on coordinate position, L_{ij} is the ray path from i to j, and N is the normalization for the volume influenced by V_n.The ray-path L_{ij} is computed in final model obtained by observation, and takes into account the real ray-path geometry.

The resolution parameters, i.e. RDE, S_j and DWS, are calculated for each parameterization, since they depends on it. In fig. S14 are shown the RDE (a), the S_jj (b) and the DWS (c) for the finest parameterization 10x10x5 m^3, and, in fig. S13, the same parameters for the coarser parameterization 16x16x7.5 m^3.

In figure 3 and 4 of the main text and in figure S5 of SM, the black contour delimitates the resolved area, i.e. the area for which the tree resolution parameters (RDE, S_j and DWS) are included in a threshold value. The threshold values of S_j and DWS are chosen in order to obtain a similar contour, binding the RDE to be higher than 0.9.

The resolution matrix and the spread function allow to state that the final model is well resolved down to 30-35 m depth.

Resistivity resolution analysis

The sensitivity map is given by the diagonal elements of J^TJ matrix and is shown in Fig. S8. Each element in the mesh contains the sum of the squared sensitivities of the data, obtained after the 5th iteration. Here, the sensitivity spatial distribution is related to the measurement configuration. Around electrodes, the sensitivity is closed to 1, which indicates a perfect accuracy. As the density of D-C measurement decreases at depth, the resolution and the sensitivity value both decline. However, the ERT cross section still shows a very good resolution up to 25-30 m depth (green and light blue in the fig. S15) whereas at the bottom corner a lower resolution is observed. Therefore, looking at this sensitivity map, it appears that the interpretation of the electrical cross-section is valid.

Figure S1

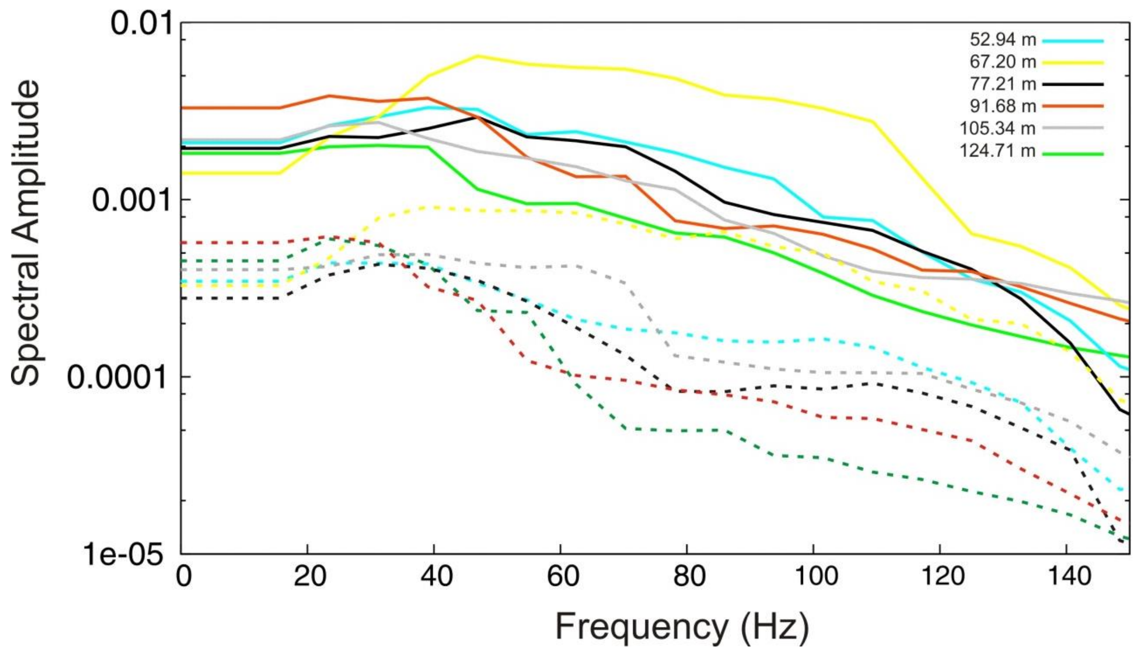

Figure S1. Spectral Amplitude as a function of the frequency for the first P wave arrival (solid lines) and pre P-wave noise (dashed lines). Different colors are associated to sources located at increasing distance as indicated by legend.

Figure S2

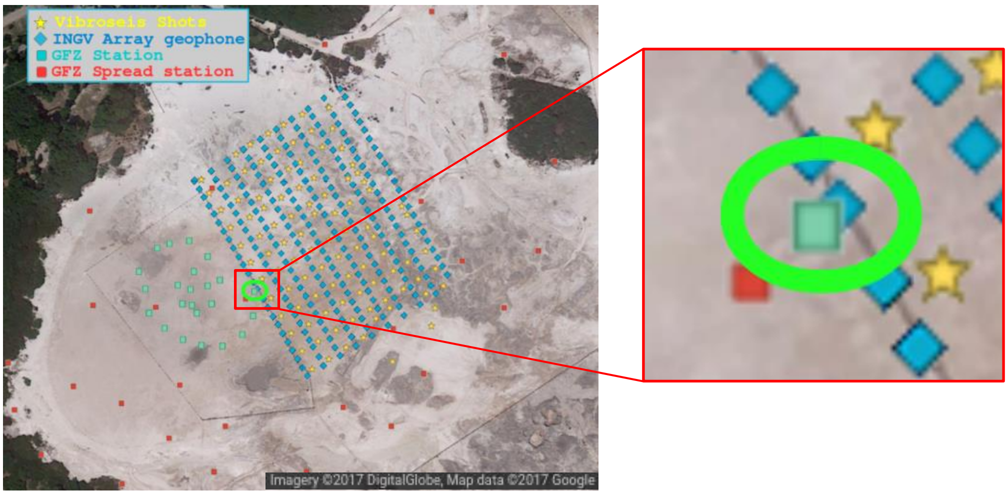

Figure S2. Station-shot configuration of the RICEN experiments. The map has been obtained with Google Maps 9.38.1 2017 (Map data: Google, DigitalGlobe): Solfatara, Pozzuoli, Metropolitan City of Naples, Italy retrieved from https://www.google.it/maps/place/Solfatara/@40.8273829,14.1385353,230m/data=!3m1!1e3!

Figure S3

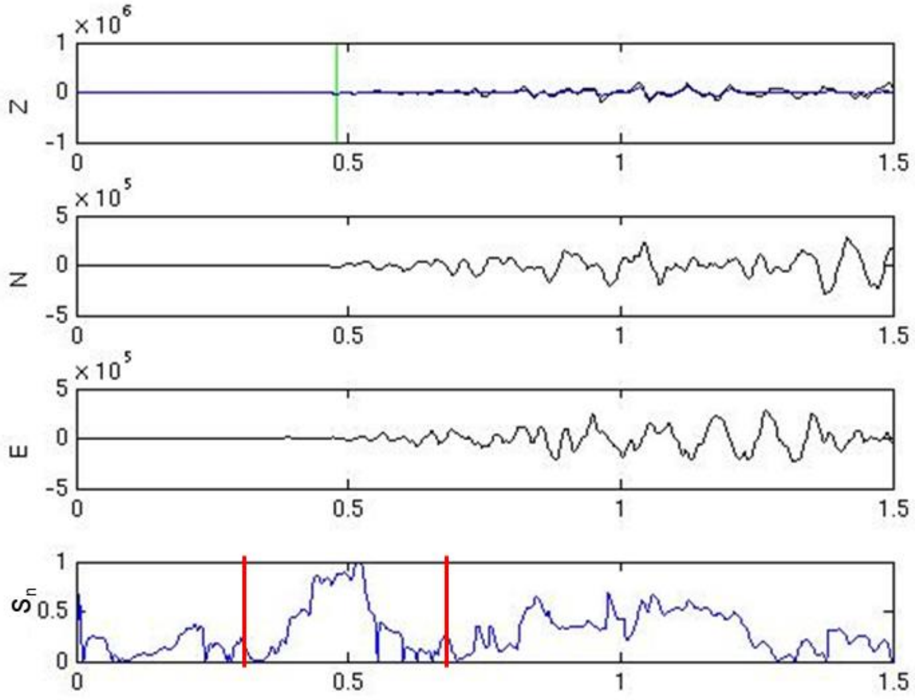

Figure S3. The Z, N and E component of waveform of shot 111 (nearest to station) and cosine of the angle (from vertical) of the direction of particle motion (sn). The green line mark the P pick, the red lines marks the time window in which was calculated the max of sn

Figure S4

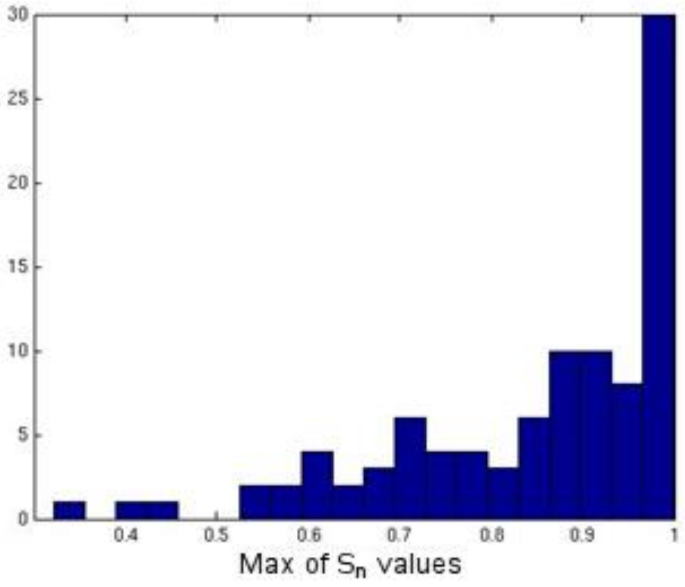

Figure S4. Histogram of sn maximum for all the 97 analyzed shots.

Figure S5

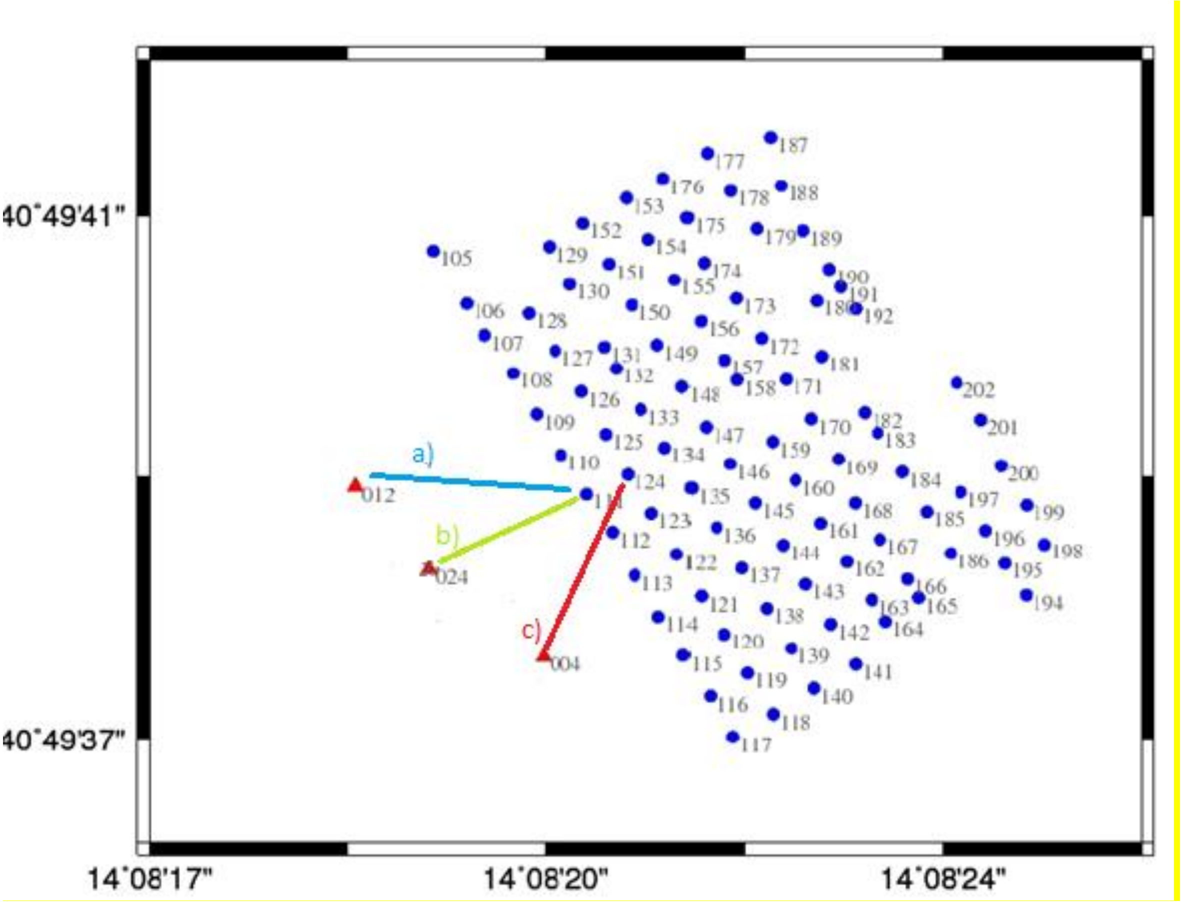

Figure S5. Station-shot configuration used in polarization analysis.

Figure S6

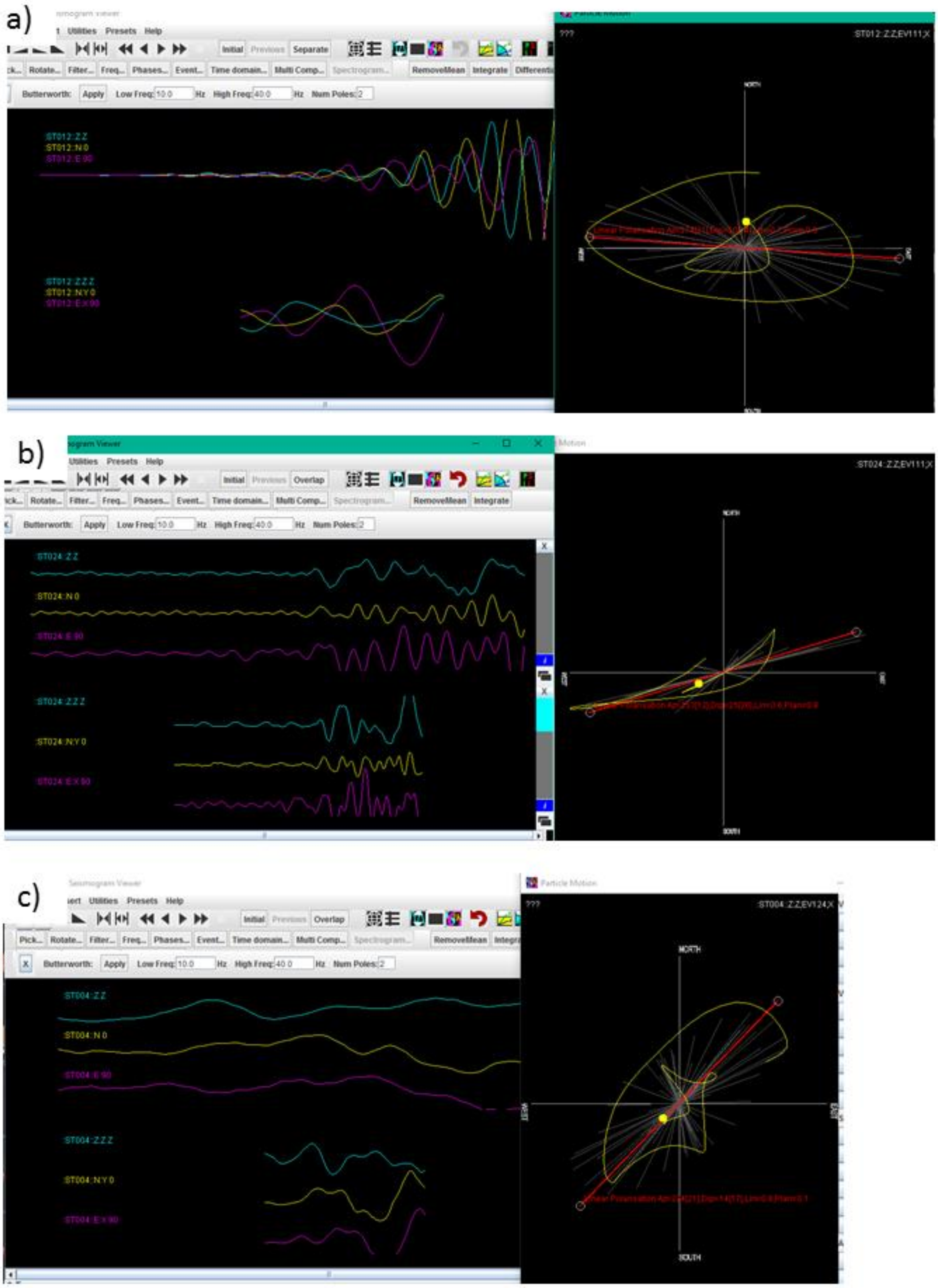

Figure S6. Polarization analysis results. The plots represents the results of polarization analysis performed on three 3-components recording corresponding to different station-source combination: a) Station 12 - Event 111; b) Station 24 – Event 111; c) Station 4 – Event 124. The left panel shows the tree-component recording and a zoom in the first arrival time window, used to perform the analysis; the right panel shows the results of polarization analysis specifying its type (linear) and the corresponding polarization attributes.

Figure S7

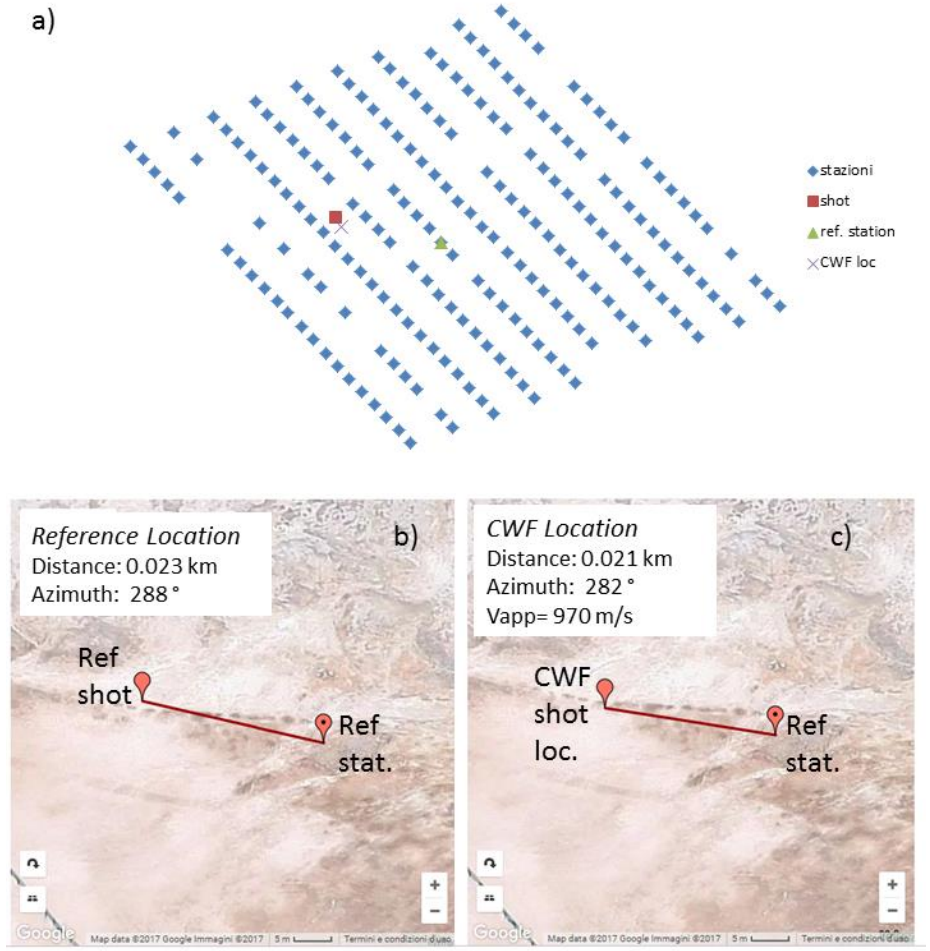

Figure S7. Result of the array location method applied at shot 132 (red square in a) recorded at 204 receivers (blu diamond in a).In panel a) the green triangle indicates the central array reference receiver and the purple " x" indicates the obtained location with the array technique. In panel b) and c) are showed, respectively, the plane view of relative position of reference shot and retrieved shot respect to the reference station. Moreover, in the label there are complete information about the distance and azimuth in the two cases. The maps in Fig. S7b-c has been obtained with Google Maps 9.38.1 2017 (Map data: Google, DigitalGlobe): Solfatara, Pozzuoli, Metropolitan City of Naples, Italy retrieved from https://www.google.it/maps/place/Solfatara/@40.8273829,14.1385353,230m/data=!3m1!1e3!

Figure S8

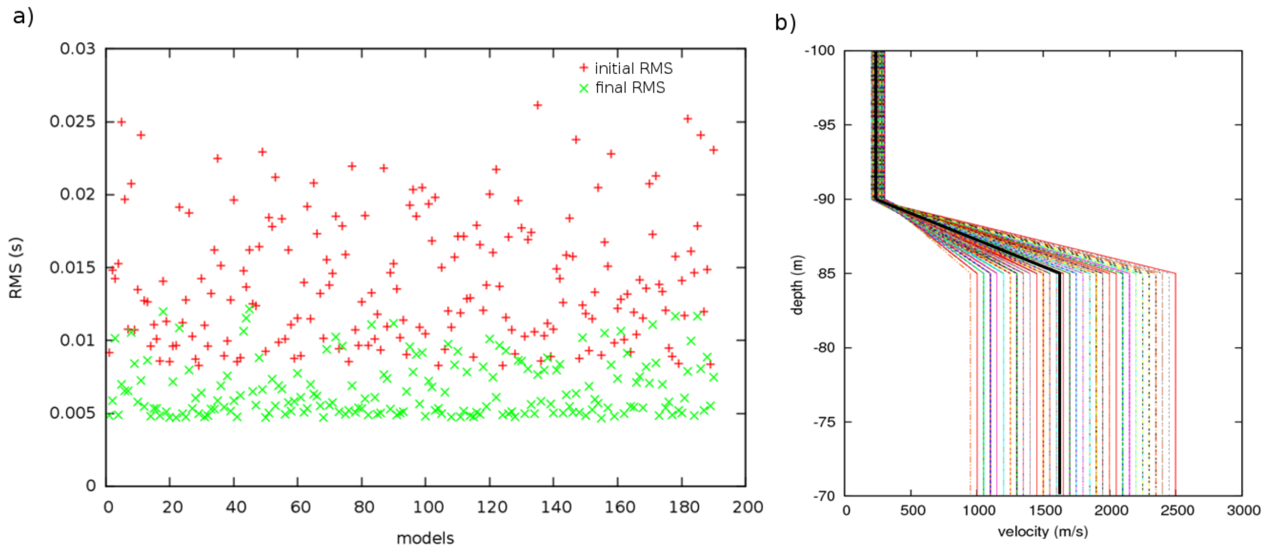

Figure S8. a) Distribution of final (green) and initial (red) rms of the inversion with the 200 2-layer velocity models. b) Plot of the 200 2-layer velocity models. The solid black line represent the model with the least final rms.

Figure S9

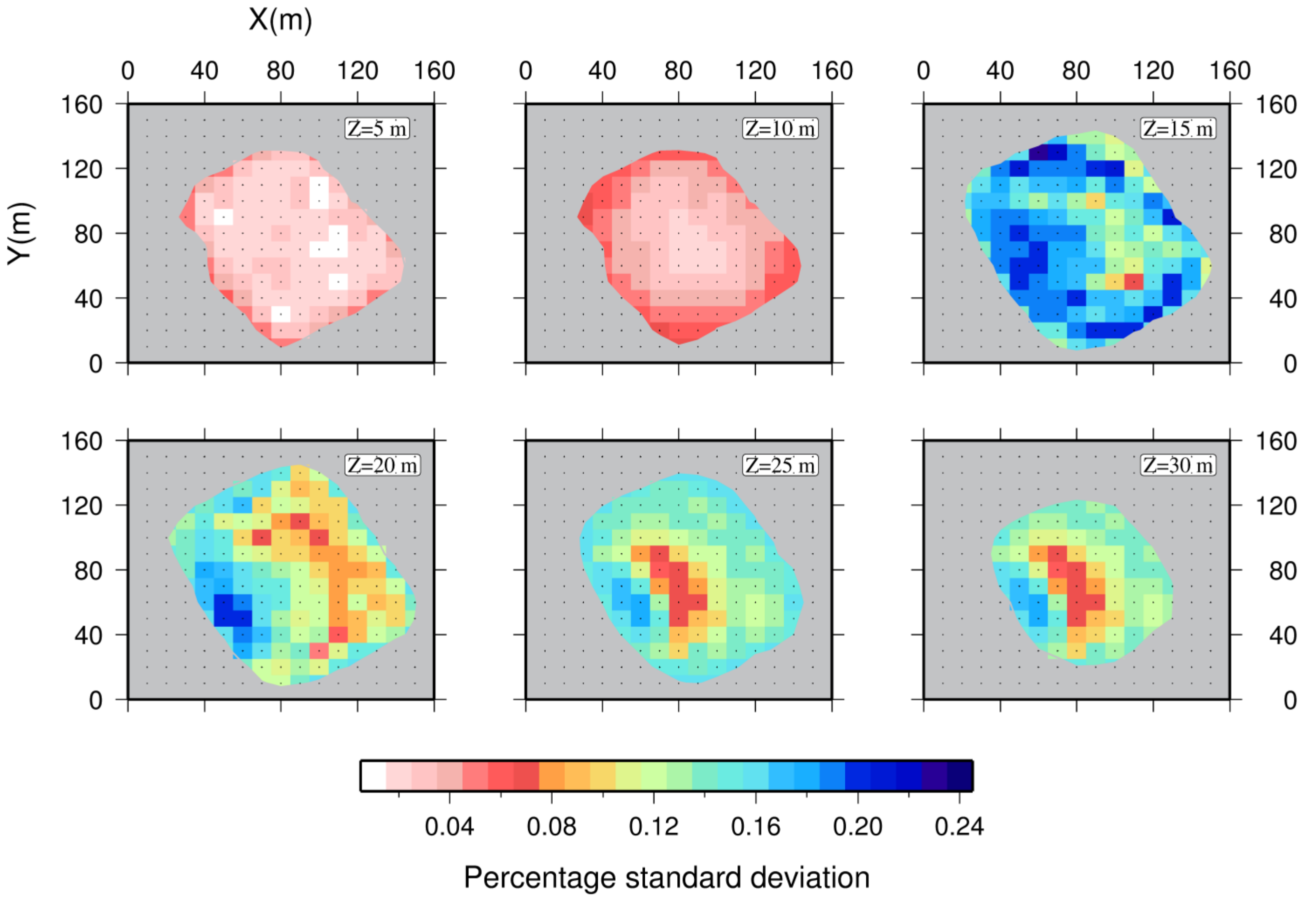

Figure S9. Map representation of the normalized standard deviation for each model parameter (σ/V_P) of the 3D velocity models obtained by the inversion with different 1D 2-layer initial velocity models. The slice are at a depth of 5 m, 10 m, 15 m, 20 m, 25 m and 30 m. The grey regions in each slice represent areas not well resolved.

Figure S10

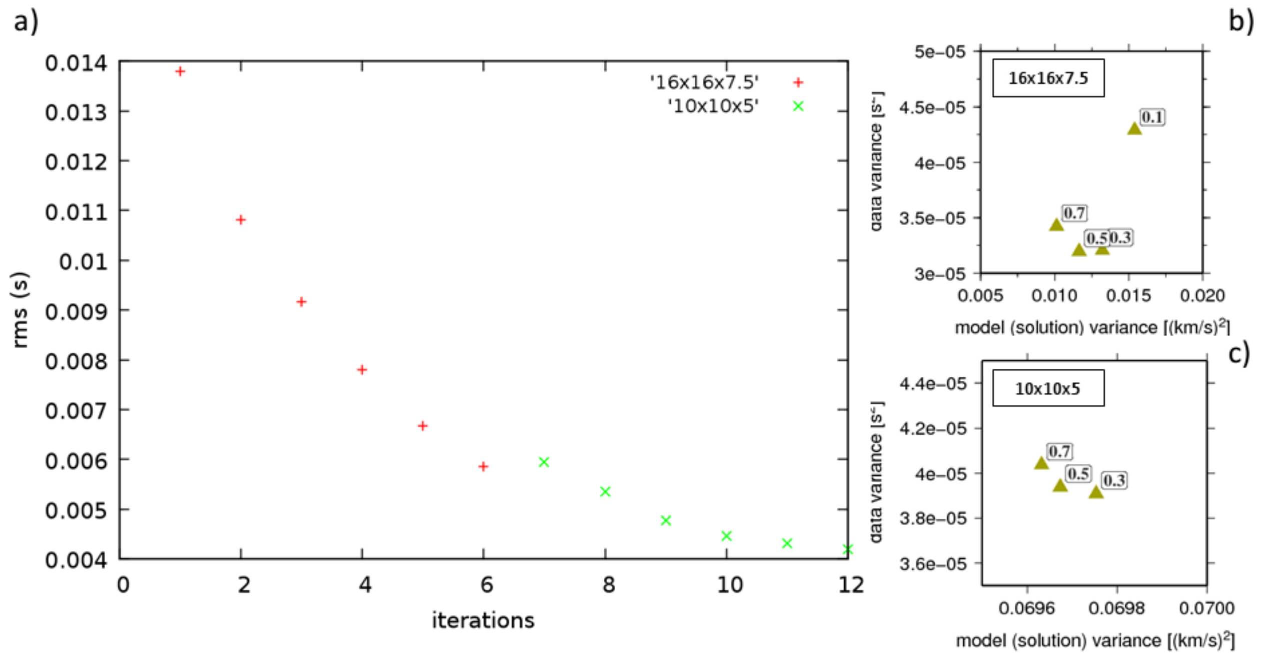

Figure S10. a) Rms curve as a function of number of iterations for the two parameterization of multiscale procedure. b-c) Trade-off curves for selecting optimal damping value for real data sets. The figures in b) and c) represent the L-curve for the inversions for V_P, respectively, in the coarse grid ($16x16x7.5m^3$) and in the finer ($10x10x5m^3$). The chosen damping for the two inversion is 0.5.

Figure S11

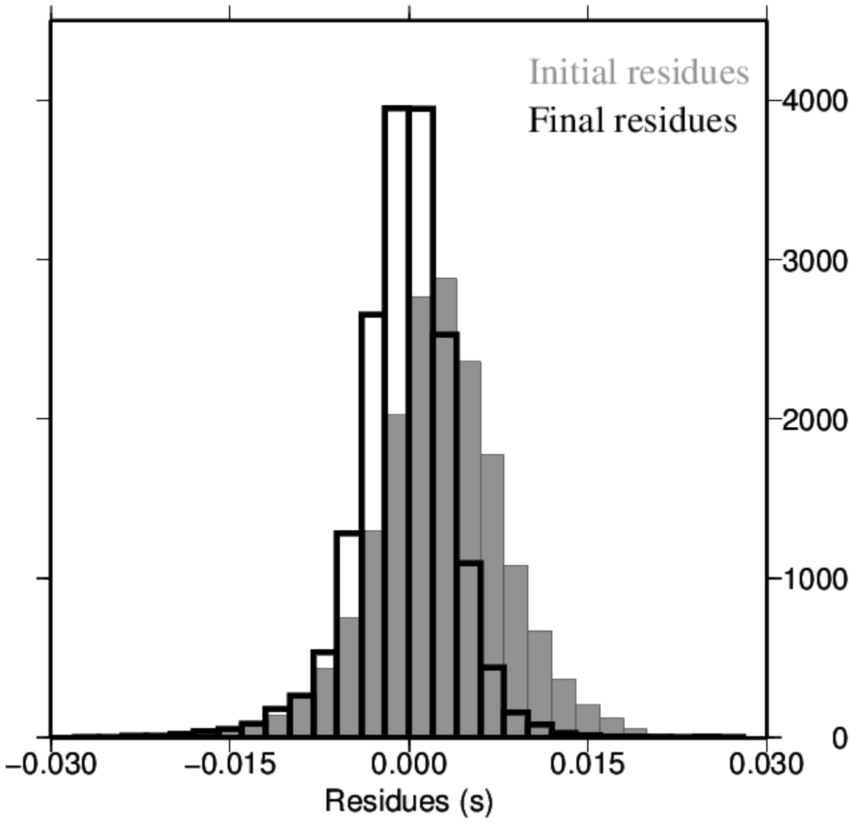

Figure S11. Distribution of inversion residuals for the finer parameterization 10x10x5. The grey histogram represents the initial residuals, the black one represents the final residuals. The final residuals histogram shows a tight distribution centered at zero.

Figure S12

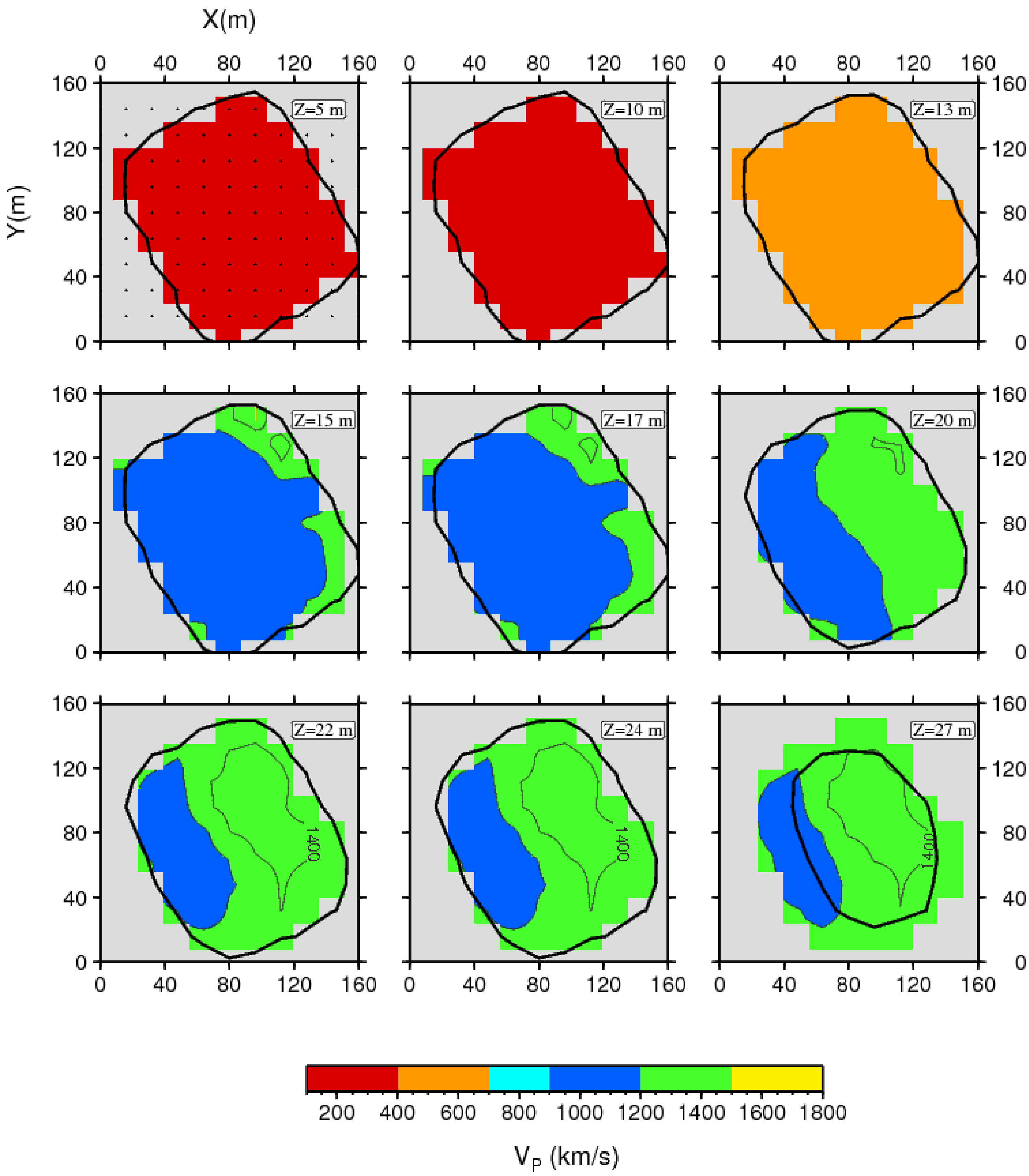

Figure S12. a) Map view at several depths of 3D velocity models obtained with the parametrization 16x16x7.5 m^3. The grey regions represent areas not covered by the rays. The black contour represents the area well the area for which the tree resolution parameters (RDE, S_j and DWS) are included in a threshold value. The model shows already the strong lateral variation of velocity values, from 20 m depth, that is than improved in the model obtained with the finer parameterization; although, overall, the velocity values are lower than the one of obtained with the parameterization 10x10x5 m^3. The resolution analysis shows that the model is well resolved up to 30 m depth.

Figure S13

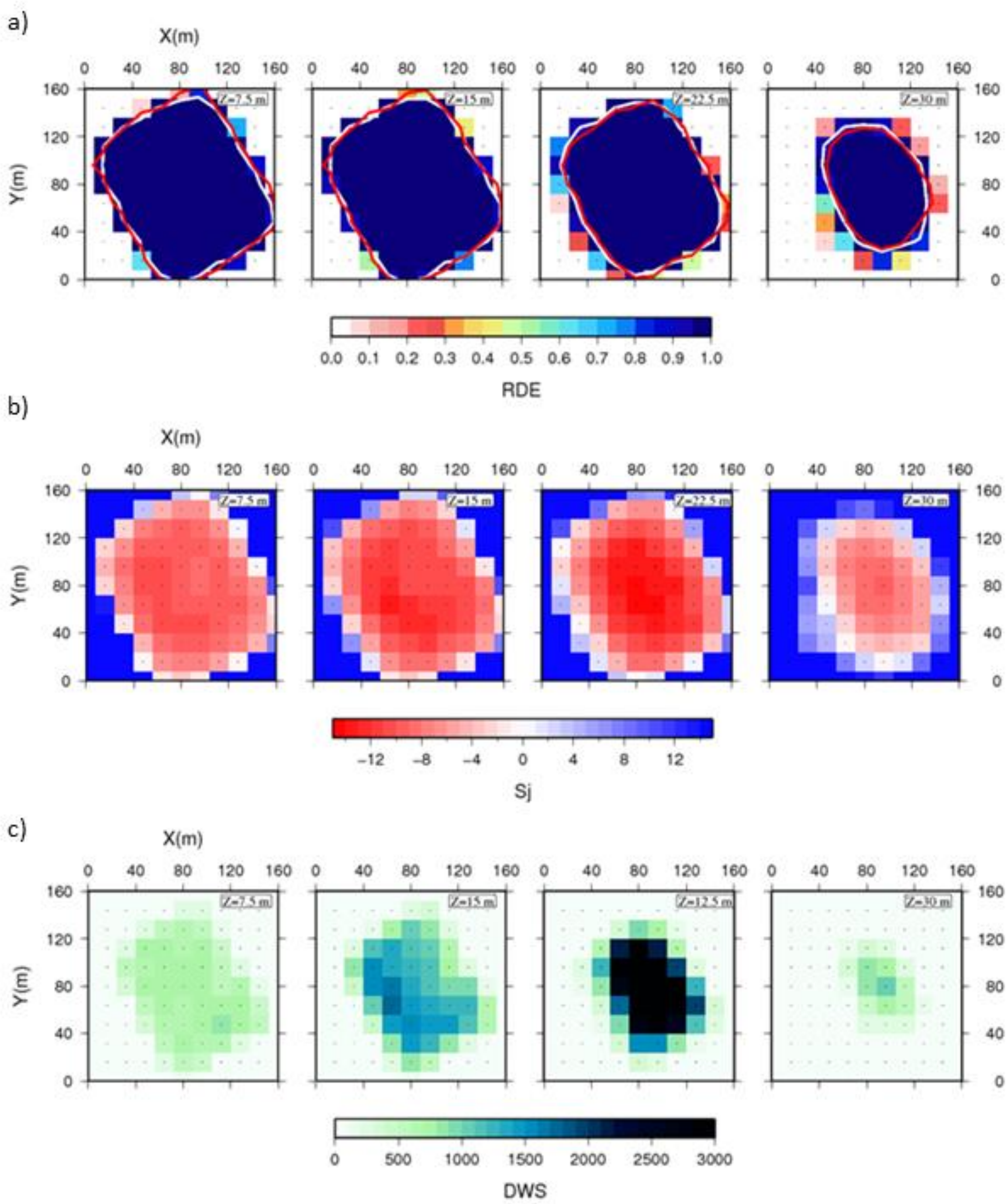

Figure S13. Resolution parameters matrix for 3D velocity model obtained with the parameterization 16x16x7.5. The full resolution matrix is represented in term of resolution diagonal element RDE (a) and spread function Sj (b) at four different depths. In panel a) the red and white contour represents the threshold values of Sj (red) and DWS (white) chosen in order to obtain a similar contour, binding the RDE to be higher than 0.9. The c) panel represents the DWS at four depths.

Figure S14

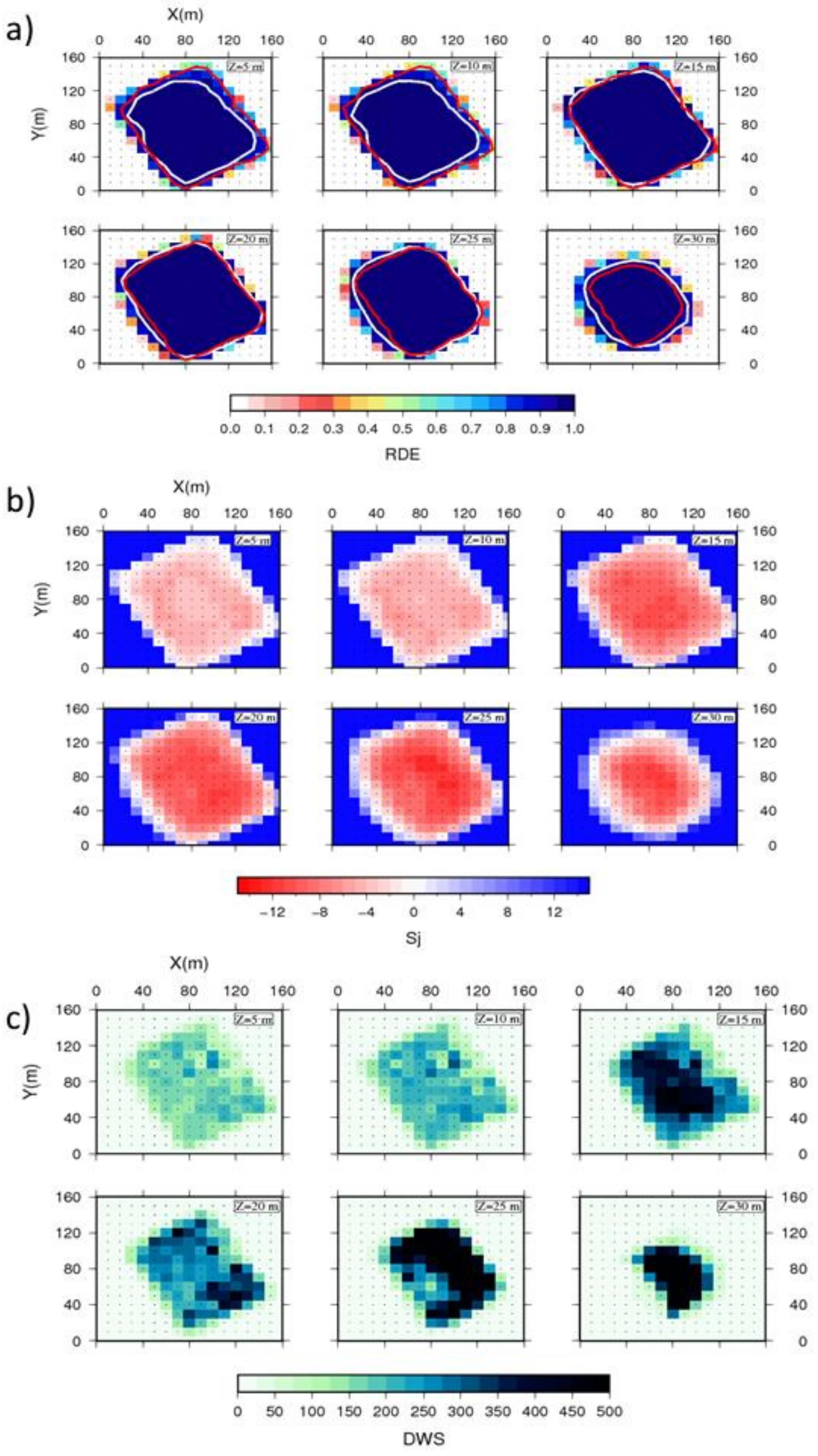

Figure S14. Resolution parameters matrix for 3D velocity model obtained with the parameterization 10x10x5. The full resolution matrix is represented in term of resolution diagonal element RDE (a) and spread function Sj (b) at four different depth. In panel a) the red and white contour represents the threshold values of Sj (red) and DWS (white) chosen in order to obtain a similar contour, binding the RDE to be higher than 0.9. The c) panel represents the DWS at different depths.

Figure S15

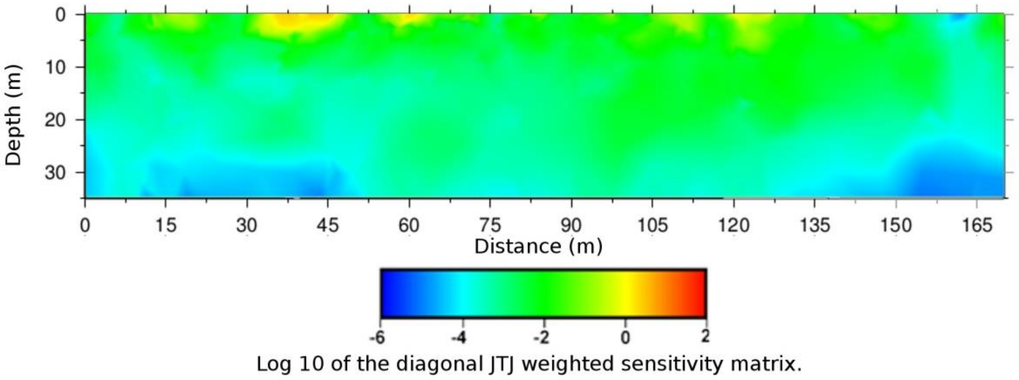

Figure S15. ERT sensitivity. The diagonal JTJ matrix shows the sensitivity of the bulk electrical conductivity values with respect to the data. This cross section shows a very good resolution up to 25-30 m depth (green and light blue in the figure) whereas at the bottom corner a lower resolution is observed.

References

1. Caudill, M. *Neural Networks Primer* (ed. Miller Freeman Publication, 1989)
2. Fahlman, S. & Lebiere., C. *The Cascade Correlation Learning Architecture*: Advances in Neural Information Processing Systems 2, 524-532 (1990).
3. Rumelhart, D.E. & McClelland, J.L (eds). Parallel Distributed Processing: Exploration in the Microstructure of Cognition, *MIT Press*.
4. Wasserman, P. D. *Neural Computing Theory and Practice:* Van Nostrand Reinhold (1989).
5. Ackermann, H. D., Pankratz, L. W. & Dansereau, D. Resolution of ambiguities of seismic refraction traveltime curves. *Geophysics*. **51**(2), 223-235 (1986).
6. Brittle K.F., Lines, L.R. & Dey, A.K. Vibroseis deconvolution: a comparison of cross-correlation and frequency-domain sweep deconvolution. *Geophysical Prospecting*. **49**, 675-686 (2001).
7. Gibson B. and Larner K. Predictive deconvolution and the zero-phase source. *Geophysics.* **49**, 379-397 (1984).
8. Cambois G. Zero-phasing the zero-phase source. *The Leading Edge.* **19**, 72-75 (2000).
9. Rosenberger, A. Real-time ground-motion analysis: distinguishing P and S arrivals in a noisy environment. *Bulletin of the Seismological Society of America*, **100**(3), 1252-1262 (2010).
10. Sissons, B. A. & Dibble, R. R. A seismic refraction experiment southeast of Ruapehu volcano. New Zealand Journal of Geology and Geophysics 24, 31-38 (1981).
11. Mora, M. et al. Shallow velocity structure and seismic site effects at Arenal volcano, Costa Rica. *Journal of Volcanology and Geothermal Research* **152**, 121-139 (2006).
12. Isaia, R. et al. Stratigraphy, structure, and volcano-tectonic evolution of Solfatara maar-diatreme (Campi Flegrei, Italy). *Geological Society of America Bulletin*, **127**, 1485-1504 (2015).
13. Montalbetti, J. F., & Kanasewich, E. R.. Enhancement of teleseismic body phases with a polarization filter. *Geophysical Journal International*, **21**(2), 119-129 (1970).
14. Almendros, J., Ibáñez, J. M., Alguacil, G., & Del Pezzo, E.. Array analysis using circular-wave-front geometry: an application to locate the nearby seismo-volcanic source. *Geophysical Journal International*, **136**(1), 159-170 (1999).
15. Rost, S., & Thomas, C.. Improving seismic resolution through array processing techniques. *Surveys in Geophysics*, **30**(4-5), 271-299 (2009).
16. Serra, M. et al. A strongly heterogeneous hydrothermal area imaged by surface waves: the case of Solfatara, Campi Flegrei, Italy. *Geophysical Journal International* **205**, 1813-1822 (2016).
17. Latorre, D. et al. A new seismic tomography of Aigion area (Gulf of Corinth, Greece) from the 1991 data set. *Geophys. J. Int*. **159,** 1013-1031 (2004).
18. Zollo, A. Bayesian estimation of 2-D P-velocity models from active seismic arrival time data: imaging of the shallow structure of Mt Vesuvius (Southern Italy). *Geophys. J. Int*. **151**, 566-582 (2002).

19. Akaike, H. A new look at the statistical model identification. *IEEE Transactions on Automatic Control.* **19**, 716–723 (1974).
20. Cavanaugh, J. E., & Shumway, R. H. A bootstrap variant of AIC for state-space model selection. *Statistica Sinica*, 473-496 (1997).
21. Rawlinson, N. & Spakman, W. On the use of sensitivity tests in seismic tomography. *Geophysical Journal International*. **205**(2), 1221-1243 (2016).
22. Michelini, A. & McEvilly, T. V. Seismological studies at Parkfield. I. Simultaneous inversion for velocity structure and hypocenters using cubic B-splines parameterization. *Bulletin of the Seismological Society of America* **81**, 524-552 (1991).
23. Toomey, D. R., & G. R. Foulger Tomographic inversion of local earthquake data from the Hengill-Grensdalur central volcano complex, Iceland, *J. Geophys. Res.*, **94**, 497–510. (1989)
24. Hauksson, E., & Shearer, P. M. Attenuation models (Q_P and Q_S) in three dimensions of the southern California crust: inferred fluid saturation at seismogenetic depths. *J. Geophys. Res*. **11**, B05302 (2006).
